# Deciphering the infrared spectrum of the hydrated proton using full-dimensional quantum dynamics

**DOI:** 10.1038/s41557-026-02209-3

**Published:** 2026-07-27

**Authors:** David Mendive-Tapia, Christoph Schran, Banshi Das, Fabien Gatti, Markus Schröder, Dominik Marx, Oriol Vendrell

**Affiliations:** 1 Theoretische Chemie, Physikalisch-Chemisches Institut, Universität Heidelberg, Heidelberg, Germany; 2 Cavendish Laboratory, Department of Physics, University of Cambridge, Cambridge, UK; 3 Lennard-Jones Centre, University of Cambridge, Cambridge, UK; 4 Lehrstuhl für Theoretische Chemie, Ruhr-Universität Bochum, Bochum, Germany; 5 Université Bourgogne Europe, Dijon, France

**Keywords:** Theoretical chemistry, Physical chemistry

## Abstract

The nature of the aqueous proton has been traditionally interpreted through two limiting structural motifs: the Zundel and Eigen cations. However, experimental infrared (IR) spectra of the solvated proton reveal a far more dynamic character, as evidenced by distinct intensity modulations within the characteristic continuum absorption band. In fact, recent ultrafast two-dimensional IR spectroscopy suggests that solvation-induced structural distortions around H_2_O⋯H^+^⋯OH_2_ motifs critically shape the IR response. Here we investigate the role of such asymmetry through full-dimensional quantum dynamics simulations of the extended Zundel complex H^+^(H_2_O)_6_, which structurally encompasses both Zundel and Eigen motifs. Systematic removal of one water molecule from the second solvation shell gradually introduces deviations from the perfectly symmetric Zundel-like complex towards Eigen-like spectral features. These results provide a direct map between the asymmetric solvation environment and the structural response of the first and second solvation shells of the aqueous proton, offering a structural and dynamical basis for understanding how this asymmetry governs proton mobility in aqueous environments.

**Figure:**
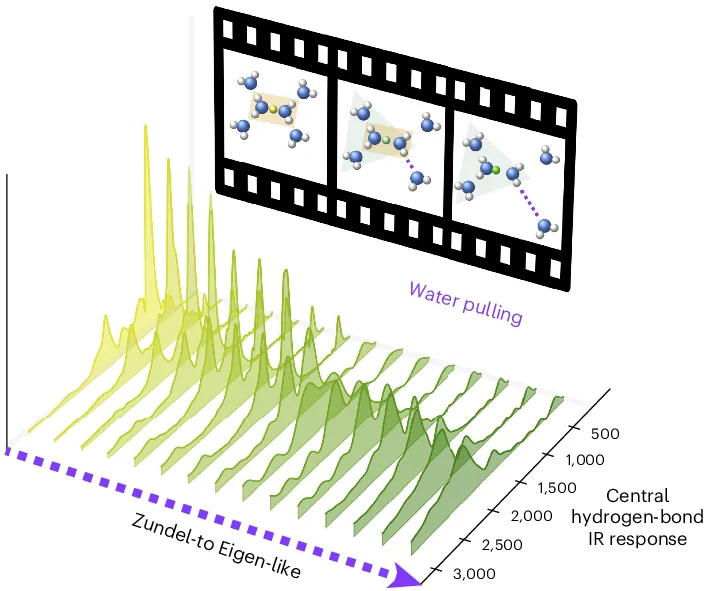

## Abstract (web summary)

The infrared spectra of the aqueous proton show dynamic character beyond the classical Zundel H^+^(H_2_O)_2_ and Eigen H^+^(H_2_O)_3_ structural motifs. Fully quantum modelling the larger H^+^(H_2_O)_6_ complex provides a direct link between asymmetric solvation environments and the spectroscopic response of the solvated proton.

## Main

Understanding the nature of the aqueous proton is of essential importance for both fundamental science^1^ and technological applications^2,3^, such as the concept of pH in acid–base chemistry^4^, proton transfer across membranes in metabolic processes^5,6^, and proton exchange in batteries for energy storage^7^. However, even though pioneering attempts to understand the hydrated proton and its transport mechanism date back more than 200 years^8,9^, consensus is still lacking on the microscopic structure and dynamics of excess protons in liquid water^10–13^.

**Fig. 1:**
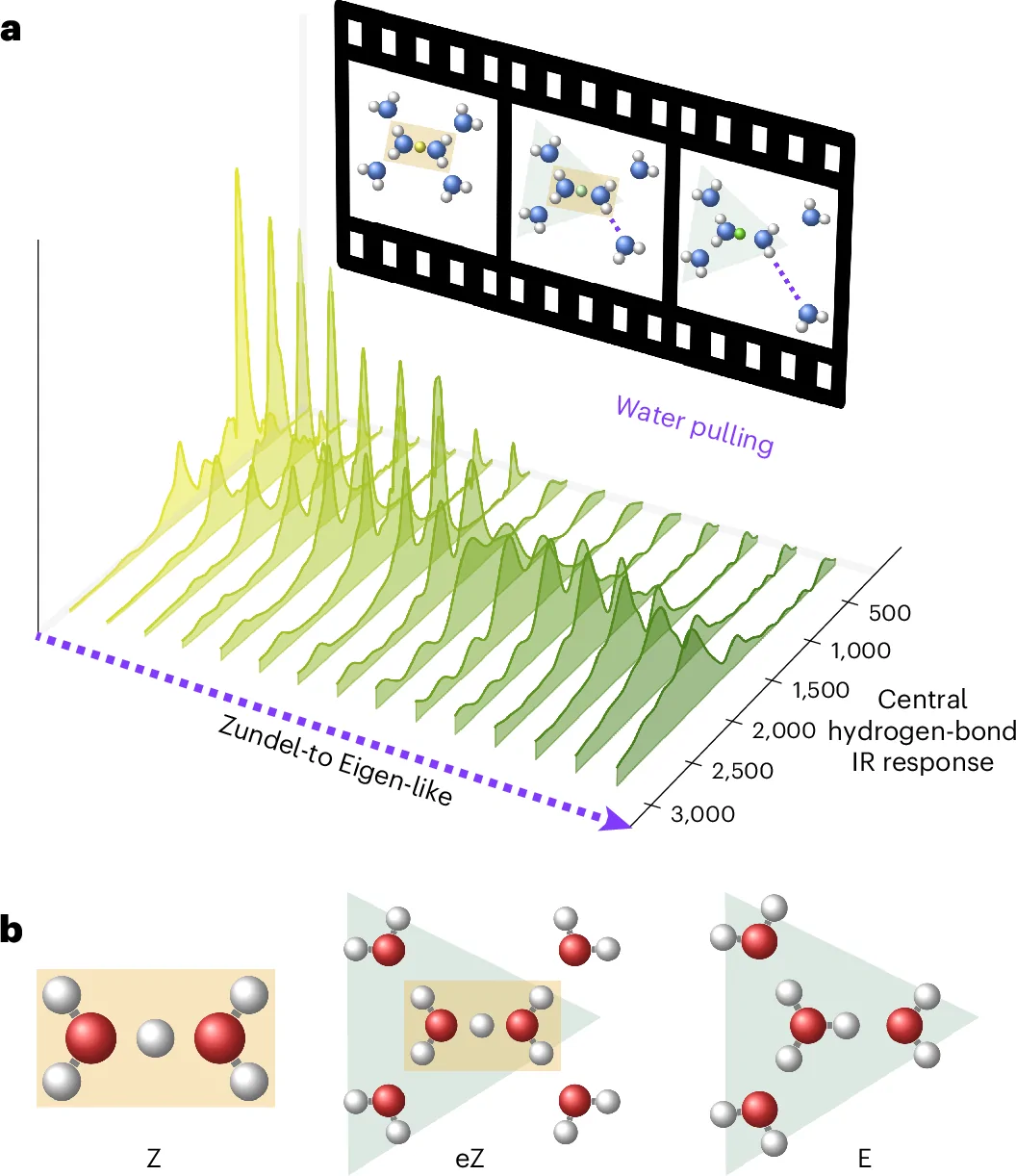
‘Stroboscopic movie’ of the local solvation environment and the corresponding IR response of the aqueous proton within the extended Zundel structure depending on its asymmetry. **a**, Schematic representation of representative structures associated with the aqueous proton within eZ as solvation asymmetry increases from left to right, together with the stroboscopic sequence of IR responses of the central hydrogen bond. The calculated rigorous quantum IR response of the central proton (see text and caption of Fig. 4), covering a width of more than 1,500 cm^−1^ as a function of asymmetry, explains the extremely broad IR response of the aqueous proton when considered in actual fluctuating environments. **b**, Molecular structures of the limiting motifs: (left to right) the Zundel (Z), extended Zundel (eZ) and Eigen (E) complexes. In the eZ complex, we denote the two water molecules of its Zundel core (H_5_O_2_^+^: orange rectangle) as the ‘inner’ water molecules and the remaining four monomers as the ‘outer’ water molecules. The eZ complex can also be viewed as an E core (H_9_O_4_^+^: green triangle) solvated by two water molecules that break the three-fold symmetry of the bare E cation; this asymmetric E-like configuration arises upon full transfer of the excess proton along the central hydrogen bond in eZ, which has also been referred to as the ‘most active proton’^24^, thus forming the nascent hydronium core, H_3_O^+^.

Two important archetypes emerged early on from attempts to characterize the very nature of a proton embedded in water^14^: the so-called Zundel (Z: H_5_O_2_^+^) and Eigen (E: H_9_O_4_^+^) cations^15,16^. In the case of the Zundel cation, the solvated proton is clamped in a symmetrical complex where there is a strong hydrogen bond between two water molecules (H_2_O⋯H^+^⋯OH_2_), whereas in the case of the the Eigen cation, a central hydronium core is hydrogen-bonded to three surrounding water molecules (H_3_O + (H_2_O)_3_). More recent computational^4,17–40^ and experimental^4,10,13,33,34,36,37,41–47^ work suggests that these structures are not mutually exclusive; rather, they represent two limiting cases^9,48,49^ with significant impact on the IR response within more complex and dynamically fluctuating local solvation environments. This has led to considerable interest in the study of the larger extended Zundel (eZ) complex^36,39,42,50–52^, H^+^(H_2_O)_6_ (compare ref. ^53^), which structurally encompasses both the Zundel (Z) and Eigen (E) motifs as schematically visualized in Fig. 1b.

**Fig. 2:**
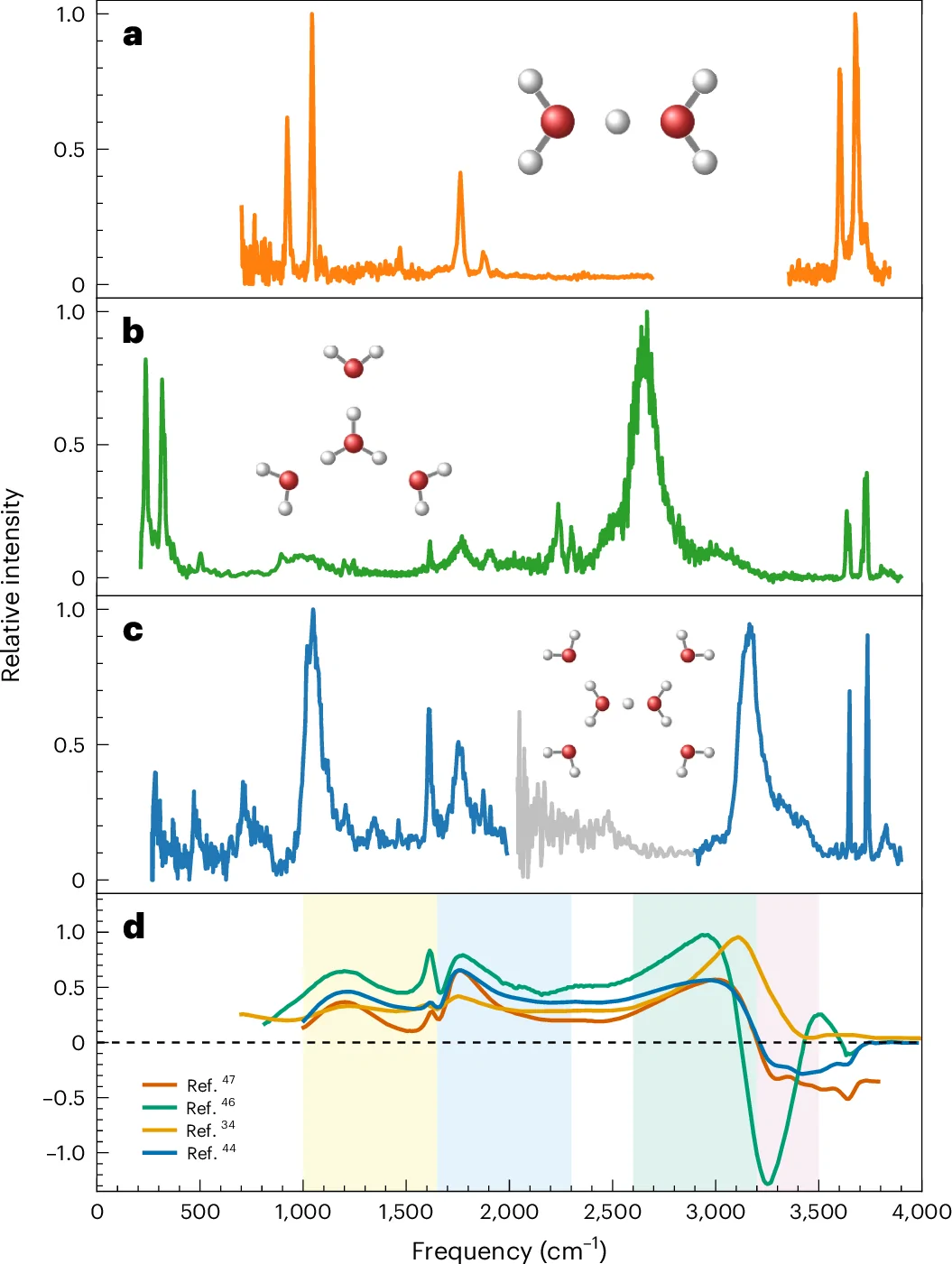
Experimental IR spectra of three protonated water clusters with increasing complex local solvation environments. **a**–**c**, Experimental IR spectra of the bare Zundel cation (Z: H_5_O_2_^+^ or H^+^(H_2_O)_2_) from ref. ^58^ (**a**), the bare Eigen cation (E: H_9_O_4_^+^ or H^+^(H_2_O)_4_) from refs. ^59,60^ (**b**) and the extended Zundel complex (eZ: H_13_O_6_^+^ or H^+^(H_2_O)_6_) from ref. ^51^ (**c**). **d**, Experimental difference IR spectra corresponding to the aqueous proton H^+^(aq) contribution in acidic solutions at various concentrations obtained by applying different subtraction schemes^34,44,46,47^ (see legend); the coloured frequency windows serve as visual guides to align and compare this panel with panels **b** and **c** in Fig. 4.

What is the reason for such ambiguity in establishing the microscopic structure of the hydrated proton? Part of the challenge lies in the inherent complexity of its infrared (IR) absorption spectrum^34,44,46,47^, which cannot be readily explained by a simple superposition of the gas-phase spectra of the limiting Z, E and eZ motifs shown in Fig. 2. Instead, it consists of a modulated continuum absorption, the broad characteristics of which extend from ~1,000 to 3,500 cm^−1^, as seen in the experimental difference IR spectra shown in Fig. 2d for various measurements and subtraction schemes^34,44,46,47^. Accordingly, the need to go beyond the normal mode picture has been explicitly demonstrated to be necessary to consistently capture the IR spectroscopic impact of interconversions between Zundel-like and Eigen-like excess proton states^4^. Furthermore, recent developments in two-dimensional (2D) IR spectroscopy^44,46,47^ have provided unprecedented access to lifetimes and vibrational couplings of the hydrated proton in solution. These reveal a complex interplay between the excess proton and its surrounding water molecules, while suggesting a prominent role of ‘asymmetric Zundel-like H_5_O_2_^+^ motifs’.

Clearly, the fundamental nature of the aqueous proton remains an open debate. Consequently, a wealth of mechanistic models have been proposed to explain the proton continuum band in the computational literature, often directly complementing experimental studies^29,31,33–38,44,47^ (see Supplementary Section 1 for an extended discussion). In this regard, a new twist to the story was recently added with the proposal of a novel three-step proton transport mechanism to explain Grotthuss diffusion and, consequently, the continuum IR absorption of the aqueous proton^39^. This idea is based on a thermal equilibrium involving two stable proton-localized structures with distinct Eigen-like and Zundel-like hydrogen-bond motifs. Notably in the stable Zundel-like hydrogen-bond motif, the excess proton is localized closer to one of the two inner water molecules, but within a larger symmetric hydrogen-bonding environment^39^. In other words, once again we find the recurrent key role of an asymmetric Zundel-like H_5_O_2_^+^ motif. In this case, the transferring proton within the most active hydrogen bond^24^—also dubbed a special bond^18^ or special pair^27^—forms a hydronium core H_3_O^+^, while the ideal Zundel structure with a centred hydrogen bond is found to be slightly unstable^39^. Nonetheless, it remains unclear how these species contribute to the observed spectral features in the aqueous proton, in particular how the local environment and asymmetry affect the IR response in the solvated H_2_O⋯H^+^⋯OH_2_ motif.

To address this, we introduce a ‘stroboscopic movie’ approach (Fig. 1a) that differs from both the ingenious IR experiments with cryogenic clusters and their associated computational modelling based on normal modes around equilibrium ‘stable’ structures, as well as from molecular dynamics-based approaches, which sample snapshot configurations from statistical ensembles that have been obtained by relying on density functional theory. Instead, we systematically desolvate the Zundel core within eZ in quasi-continuous steps (Methods). This is particularly relevant to the problem at hand, as it directly provides information on the exact nature of the motions underlying IR resonances of excess protons in aqueous environments—resolved as a quasi-continuum of stroboscopic frames—as they evolve through a whole range of configurations (see the filmstrip inset in Fig. 1a).

To achieve this, we use state-of-the-art full-dimensional quantum dynamics simulations (51 fully coupled anharmonic degrees of freedom) in combination with highly accurate potential energy and dipole moment surfaces of the eZ cation. The excellent agreement between the calculated and experimental spectra of unperturbed eZ in the full spectral range validates the model’s predictive power. Further analysis of the resulting molecular quantum dynamics—for both the equilibrium and distorted cases—allows us to assign the observed IR peaks and reveal the corresponding coupled motions within the local solvation environment. Notably, we find that when gradually introducing asymmetry into the second solvation shell around the Zundel core, one observes a seamless transition from the IR responses of the perfectly symmetric Zundel complex to the signatures of asymmetrically hydrogen-bonded Zundel motifs, and up to Eigen-like patterns of the hydrated proton (Fig. 1a). Importantly, tracking these gradual changes enables us to link the IR spectrum of the asymmetric eZ complex to the experimental spectra of the aqueous proton, thereby providing an accurate spectroscopic model that connects the observed continuum absorption band with the micro-solvation structure of the Zundel core (Fig. 1b).

## Results

### IR spectrum of the extended Zundel complex

**Fig. 3:**
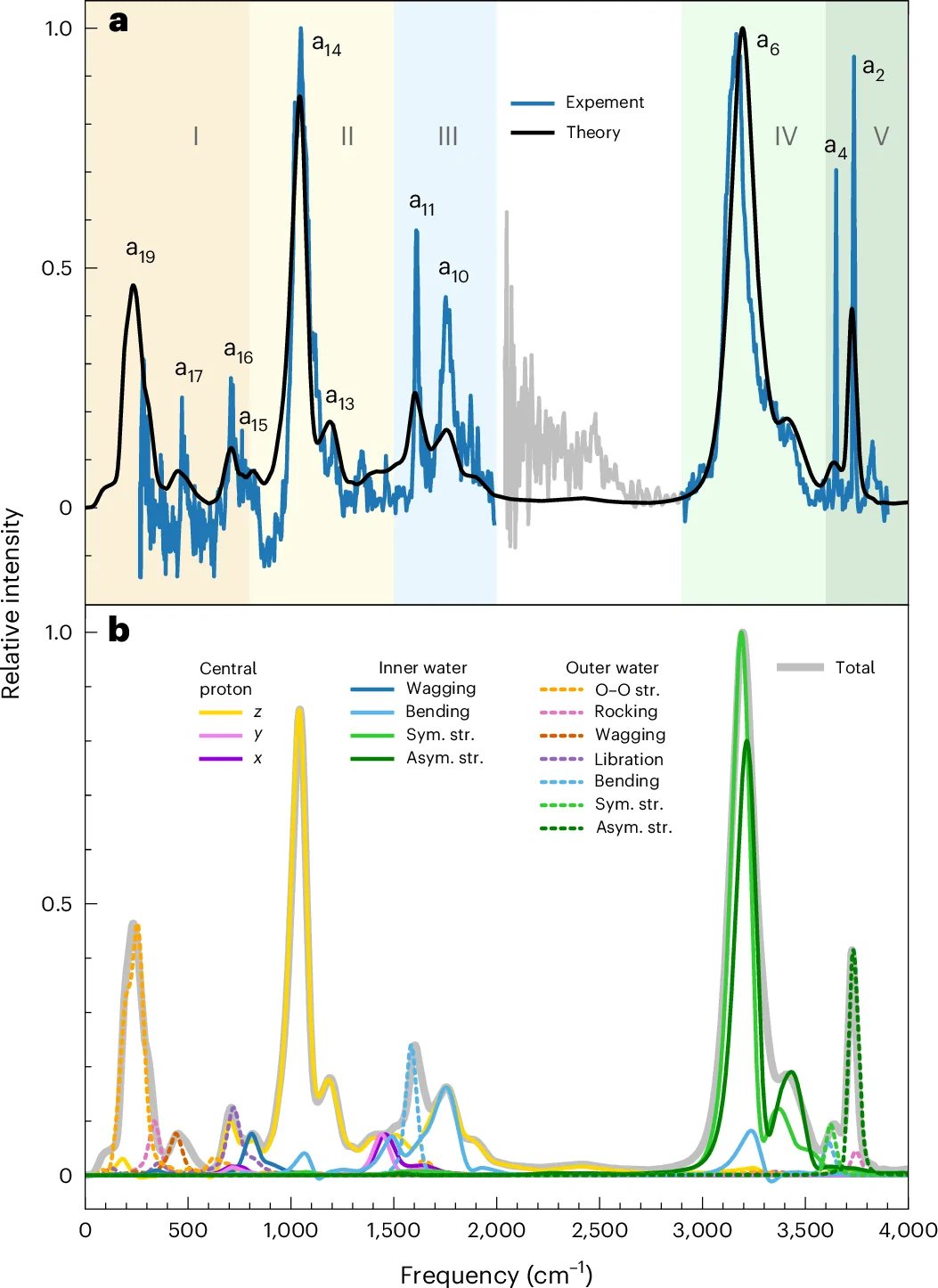
IR spectrum of the extended Zundel complex in the gas phase. **a**, Calculated linear IR absorption spectrum (black) and experimental vibrational spectrum from IR^2^MS^2^ spectroscopy^51^ (blue) of the protonated water hexamer H^+^(H_2_O)_6_; the grey line shades out an ill-resolved frequency window given the flat 2,000–3,000 cm^−1^ baseline reported in fig. 5e of ref. ^61^ based on Ar tagging spectroscopy. The labelling of the simulated spectrum indicates the main groups of bands by roman numbers (I, II, …) and corresponding subbands by letters (a_1_, a_2_, …). **b**, Decomposition of the full spectrum (grey) from **a** using localized vibrational excitations as well as the assignment of the most relevant peaks in terms of vibrational motion (colours, inset) as detailed in the text.

The calculated IR spectrum of the eZ complex is shown in Fig. 3a, together with the experimental spectrum reported in ref. ^51^ that was achieved via isomer-selective double-resonance population labelling spectroscopy in messenger-tagged H^+^(H_2_O)_6_·H_2_ complexes, implying that experimental intensities might differ from those of the linear absorption spectrum. Meanwhile, the total spectrum is decomposed according to localized excitations of specific coordinates in Fig. 3b. Every excitation produces a spectral fingerprint (depicted in a different colour and type of line), which is then superimposed on the dipole spectrum. Broadly, we divide the spectrum into five distinct spectral regions, I to V.

Region I, below 800 cm^−1^, is dominated by slow and highly anharmonic modes of the outer water molecules, namely the angular displacements (wagging, rocking) of the outer water molecules and the stretch vibration of the outer oxygen–oxygen atoms. In particular, band a_19_, centred at ~220 cm^−1^, corresponds to the latter, and lies almost completely below the experimental threshold. Region II features strong absorption near 1,000 cm^−1^, bands a_14_ and a_13_, caused by the response of the proton transfer mode along the central hydrogen bond (*z* direction: yellow solid line in Fig. 3b). In contrast, the motion of the central proton perpendicular to the O–O axis (*x*, *y* directions: blue and turquoise solid lines) presents a comparatively much weaker response due to a smaller modulation of the dipole moment. The proton transfer fingerprint (solid yellow) overlaps with the dipole spectrum in region II, and also contributes to band a_10_ and its 1,600–1,800 cm^−1^ shoulder in region III. In particular, a_10_ emerges when either the proton transfer mode or the asymmetric bending of the inner water molecules is excited (solid blue), indicating a strong coupling between these two modes^54^. The shoulder of this band also appears in the IR spectrum of the bare Zundel core and corresponds to an overtone of the proton transfer mode^55^. As discussed later, the coupling between proton transfer and bending modes plays a prominent role in determining the response of the excess proton above 1,700 cm^−1^ in asymmetric eZ configurations. By contrast, the sharp absorption at 1,618 cm^−1^ (a_11_) is not directly coupled to the proton transfer mode; rather, it corresponds to the bending mode response of the second shell of outer water molecules at a similar frequency to this vibration mode in isolated water.

Region IV, above the empty spectral region between 2,000 and 3,000 cm^−1^, presents a broad band structure with subbands (a_6_) and a shoulder, both originating from OH-stretching vibrations of the inner water molecules forming the Zundel core motif. This band is ~500 cm^−1^ wide, similarly to the hydronium band of the Eigen complex^56^ located at ~2,700 cm^−1^. This is indicative of a strong coupling of the inner OH-stretch vibrations with anharmonic angular displacements of the scaffold of this complex^56^. Hence, this broad response suggests that the hydrogen bonds connecting the inner and outer water molecules are highly active and can easily become the most active hydrogen bond due to fluctuations, when embedded in the bulk^39^.

Last, the broad band response of the inner stretch vibrations contrasts with the sharper response of the stretch vibrations of the outer water molecules in region V, both in the calculated and experimental spectra, indicative of the weaker couplings of the dangling hydrogen atom vibrations with the rest of the cluster. It is noteworthy that the a_4_ band displays a narrower and more defined peak in the experimental spectra when compared to theory. A similar difference between simulated and experimental bandwidths was found in the IR spectrum of the Eigen complex^56^. It thus can be attributed to the stronger correlation between the locally symmetric stretching modes of the outer water molecules and the rest of the complex, as opposed to the more decoupled, locally asymmetric stretch modes.

### Local solvation of the bare Zundel cation destroys the Fermi resonance doublet

**Extended Data Fig. 1:**
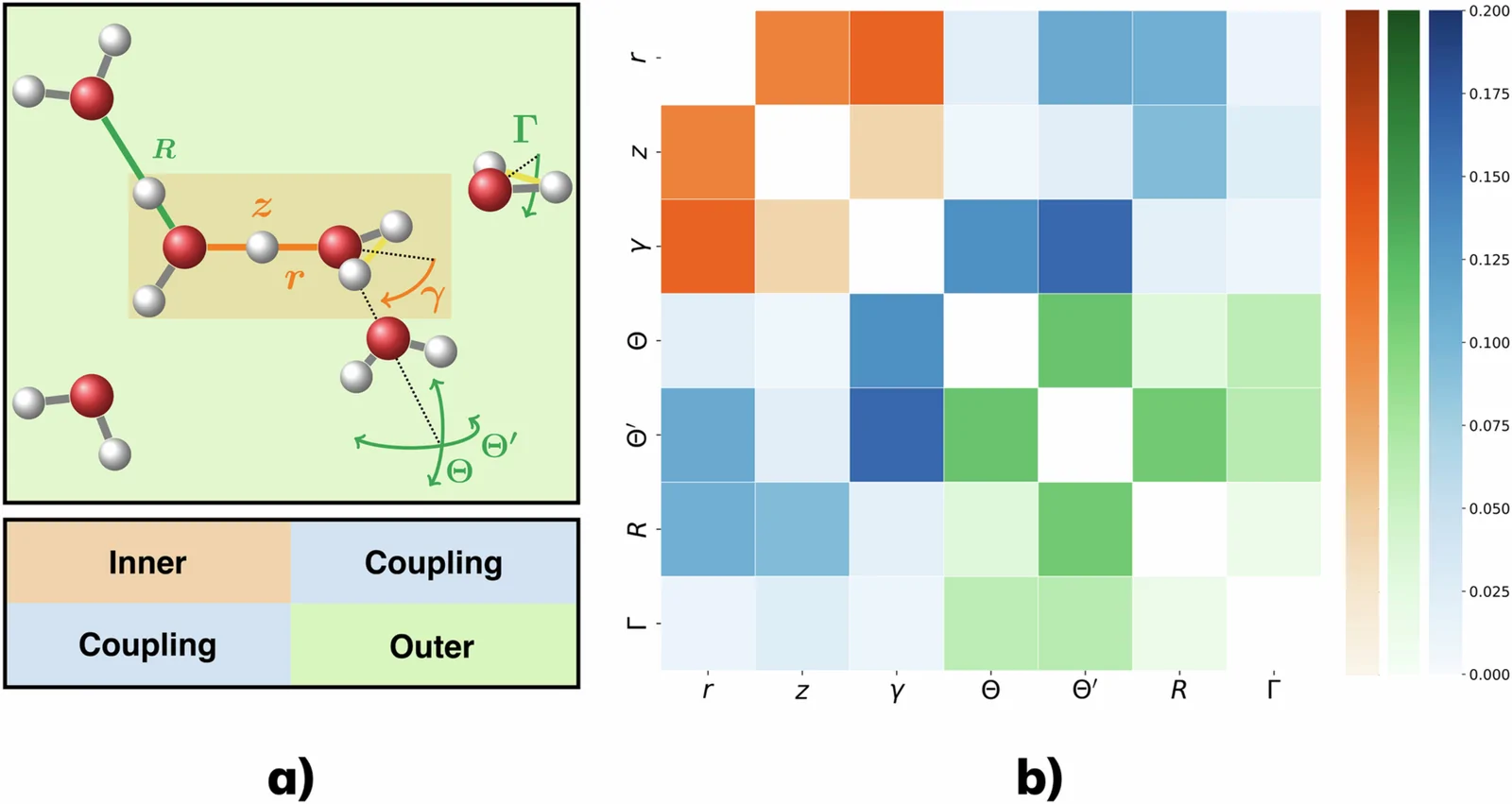
Impact of local solvation effects on the Fermi coupling in the extended Zundel complex. **a**) Coordinate definitions within a sketch of the extended Zundel complex: From left to right these are the outer-layer oxygen–oxygen distance (*R*), central proton coordinate (*z*), inner-layer oxygen–oxygen distance (*r*), inner-layer wagging (*γ*), outer-layer water librations (*Θ* and $\Theta {\prime}$), and outer-layer wagging (*Γ*). **b**) Reduced-dimensionality correlations between the most important coordinates involved in the Fermi resonance of the Zundel core and a selected subset of coordinates from the second solvation shell. We refer to Supplemental Section S3 for details on how the strongly coupled sub-blocks were combined based on the full-dimensional correlation matrix. Lowercase notation is used for motions involving the Zundel core, while uppercase indicates motions associated with the solvation environment. Four equivalent sets of uppercase coordinates exist, one for each outer water molecule, but only one coordinate of each set is shown for clarity.

One remarkable difference between the experimental IR spectrum of eZ and the bare Zundel cation is the absence of the characteristic Fermi resonance of the latter^57^; when comparing the spectra in Fig. 2a,c a broad unimodal peak appears, replacing the sharp doublet near 1,000 cm^−1^. In the bare Zundel cation, this hallmark arises from an energy shift and intensity borrowing effect between the fundamental of the central proton coordinate along the hydrogen bond of H^+^(H_2_O)_2_, and the overtone of the low-frequency wagging with the O–O stretching motions (Extended Data Fig. 1a), which incidentally have very similar frequencies^57^. Therefore, because the central structural motif H_2_O⋯H^+^⋯OH_2_ is preserved as the core of H^+^(H_2_O)_6_, upon the addition of four water molecules to H^+^(H_2_O)_2_, the following question arises: is the prominent proton transfer doublet masked due to a spectral broadening effect or is the coupling mechanism of the Fermi resonance itself destroyed due to the impact of local solvation effects?

**Fig. 4:**
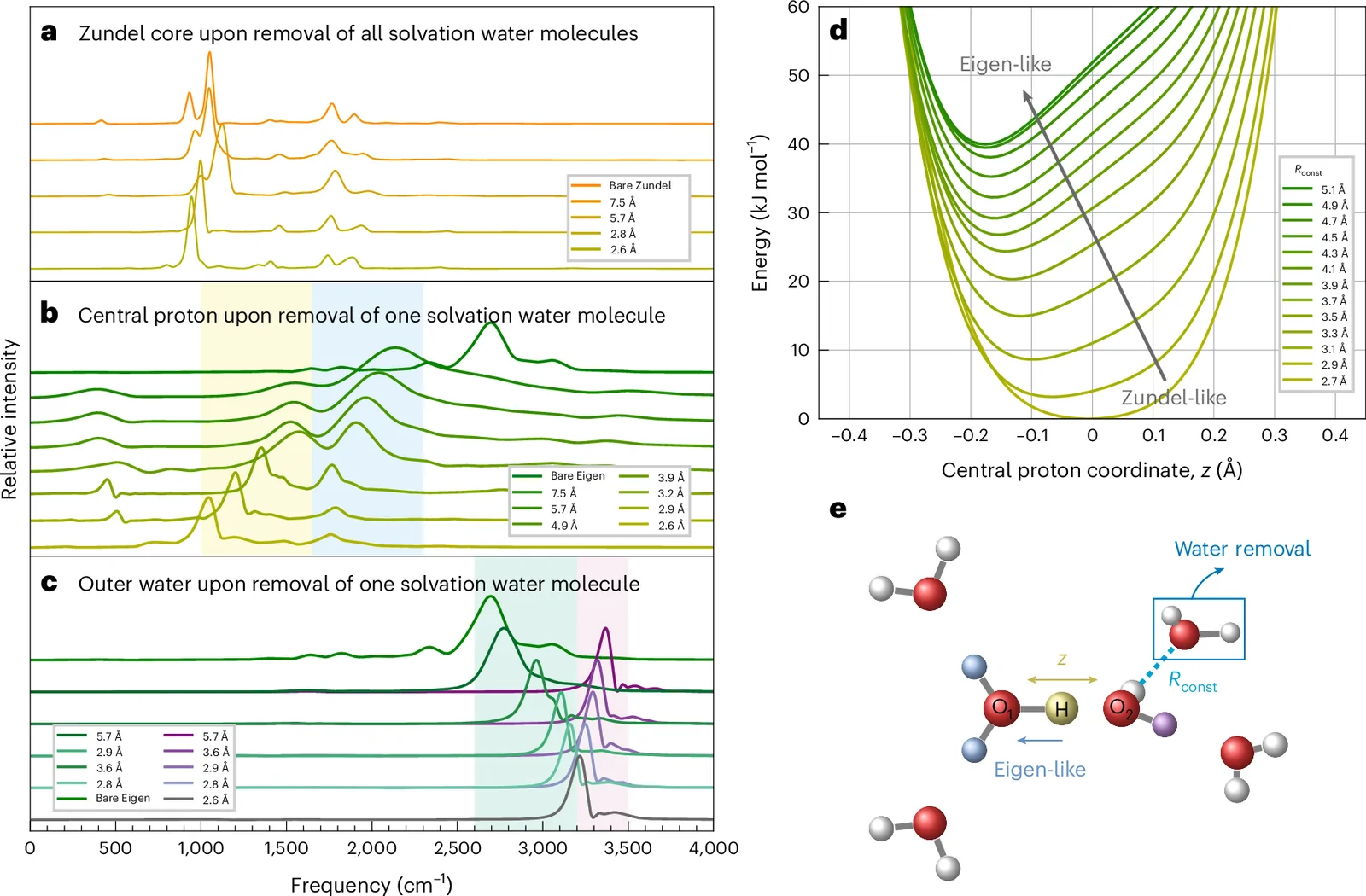
Spectral response of the extended Zundel complex as a function of the structure of the local solvation environment. **a**, Response upon excitation of the central proton hydrogen bond as a function of the symmetric detachment of all four solvating water molecules. The topmost spectrum (orange) provides the reference given by the bare Zundel cation H^+^(H_2_O)_2_. **b**, Response upon excitation of the central proton (large yellow proton in **e**) as a function of the asymmetric removal of a single solvating water molecule (upper right in the blue box in **e**). The topmost spectrum (green) provides the reference given by the bare Eigen cation H_3_O + (H_2_O)_3_ from ref. ^56^. **c**, Response upon excitation of the outer protons along the hydrogen bonds to the three remaining outer water molecules upon asymmetric removal of one solvating molecule, as done in **b**. The coloured frequency windows in **b** and **c** highlight similarities to the experimental IR response shown in Fig. 2d and are discussed in the text. **d**, Potential energy profile along the central proton coordinate (*z* as defined in **e**) for the asymmetric removal of one water molecule, as done in **b** and **c**, according to the neural network-based CCSD(T)-quality potential energy surface (Supplementary Section 2); note that 2*z* is equal to the usual $\delta ={d}_{{{\rm{O}}}_{2}{\rm{H}}}-{d}_{{{\rm{O}}}_{1}{\rm{H}}}$ coordinate for collinear O_1_⋯H⋯O_2_ structural motifs. **e**, Illustration of the removal of the outer water molecule in the blue box upon extension of *R*_const_ as visualized by the blue dotted line.

The key to answering this lies in rigorously deconstructing the effects of the second solvation shell. We addressed this by desolvating the Zundel core within eZ by gradual removal of all four solvating water molecules in a perfectly symmetric way. The spectra corresponding to the response of *z* as a function of the four equal constraint distances (${R}_{{\rm{const}}}$; Methods) are compiled in Fig. 4a. Clearly, the Fermi doublet emerges progressively as the solvating water molecules are pulled away from 2.6 to 7.5 Å (yellow to orange), closely matching the doublet feature observed in the spectrum of the bare Zundel cation (uppermost spectrum).

Further insight is gained from a deeper analysis of the frequency shifts derived from the outer-layer micro-solvation. In the Z cation, the fundamental of the wagging mode appears at ~100 cm^−1^, with the overtone responsible for the coupling with *z* appearing at 378 cm^−1^ with much lower intensity^55^. This is in clear contrast to the eZ complex, where the inner water wagging motion appears just above 800 cm^−1^ (Fig. 3b); thus, the micro-solvation of the Zundel core leads to a frequency shift of ~400 cm^−1^ in the corresponding wagging modes in eZ. Further analysis of the many-particle nuclear density distribution derived from our potential energy surface of eZ confirmed that the prominent Fermi resonance that characterizes the Zundel motif is destroyed due to hindered wagging motion of the inner water molecules, even in a perfectly symmetric local hydrogen-bond environment surrounding the Zundel core H^+^(H_2_O)_2_ (Extended Data Fig. 1b). In contrast, solvation effects on the *ungerade* bending mode of the inner water molecules are much weaker, and the frequency of this mode is almost unchanged between the bare Zundel core and the eZ complex (Methods).

### Dissecting spectral fingerprints of the aqueous proton within the extended Zundel complex

**Extended Data Fig. 2:**
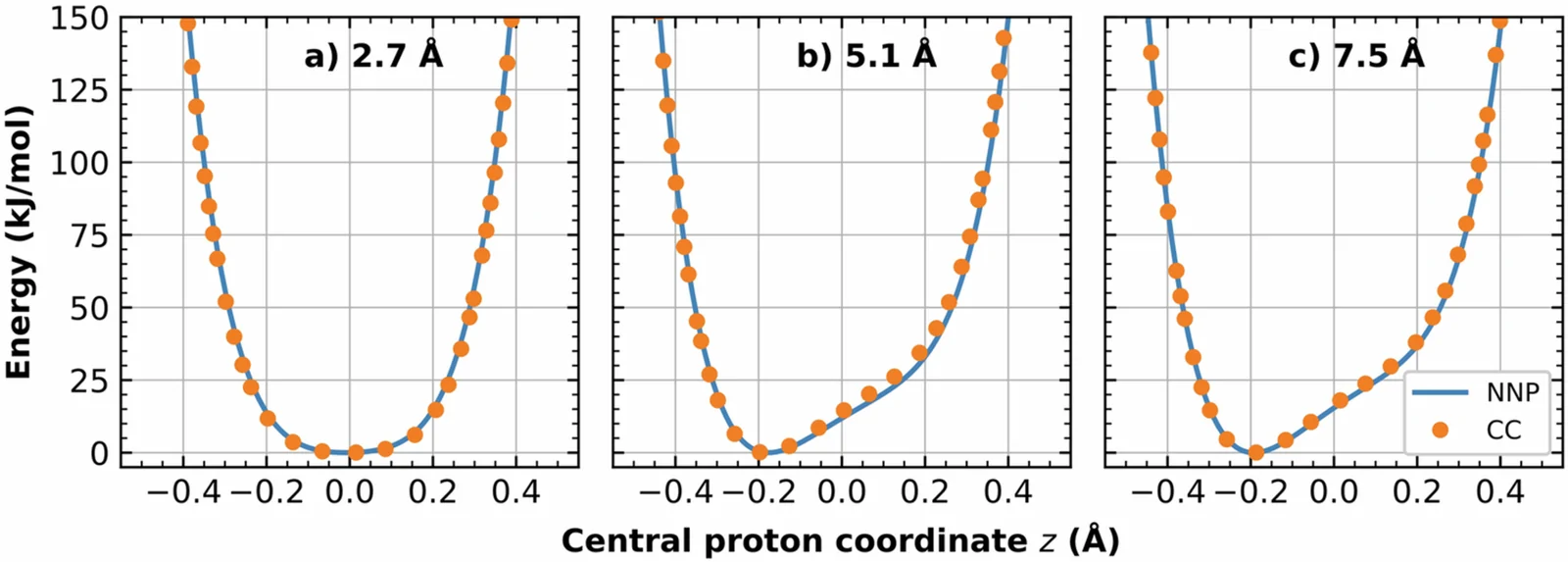
Scans of the potential energy profile along the central proton coordinate *z*. Our neural network potential (NNP) is compared to the coupled cluster (CC) reference data (marked with circles) for three representative cases of the asymmetric removal of one outer water molecule: **a**) 2.7 Å, **b**) 5.1 Å, **c**) 7.5 Å. All energies are shown relative to the respective minimum energy in each potential energy scan.

Although the previous analysis offers deep insights into solvation effects on the IR response of the Zundel core, such symmetric desolvation of the Zundel core cannot be related to the local fluctuating asymmetries experienced by excess protons in the liquid state. In the following, we thus address the effects of the asymmetric removal of a single solvation water molecule; henceforth *R*_const_ now refers to the constrained oxygen–oxygen distance of one, rather than four water molecules. This results in a dramatic change in the shape of the potential energy profile of the central proton hydrogen bond, which is illustrated in Fig. 4d with a series of energy cuts along the central proton coordinate (*z*) for fixed values of *R*_const_ ranging from 2.7 to 5.1 Å. In other words, the initially centred proton in H_2_O⋯H^+^⋯OH_2_ is progressively driven towards a hydrogen-bonded H_3_O^+^ Eigen-like motif (see Methods and Extended Data Fig. 2 for more details).

As one of the outer water molecules is gradually pulled out of the eZ complex, the fully quantum and 50D (full dimensional minus the constrained coordinate) simulations of the central proton response reveal the appearance of a doublet structure wandering through the spectral region between 1,500 and 2,300 cm^−1^ (Fig. 4b). This doublet structure reaches equal intensity of both peaks for *R*_const_ ≈ 3.9 Å, when the middle point between them is located at ~1,750 cm^−1^, in almost perfect alignment with the maximum of the broad band in the blue-shaded frequency window of the experimental aqueous proton spectra in Fig. 2d. For larger asymmetry, *R*_const_ > 5 Å, this structure morphs into a band similar to the response of the excess proton in the bare Eigen complex, but redshifted (compare the topmost bare Eigen response (green) with the response at *R*_const_ = 7.5 Å in Fig. 4b). The IR response in Fig. 4b due to the excited yellow-coloured proton in Fig. 4e never reaches a perfect Eigen response due to the presence of the solvating water molecule still attached to the purple-coloured proton (Fig. 4e).

Finally, we turn to the IR response of the outer hydrogen bonds for the symmetric solvation environment (Fig. 4c). They display a main peak corresponding to the band at ~3,200 cm^−1^ in the full spectrum (corresponding to an *R*_const_ distance of 2.6 Å). Symmetry breaking due to increasing *R*_const_ now leads to two types of hydrogen bond, indicated by green and purple hydrogen atoms in Fig. 4e. The two protons of the emerging H_3_O^+^ core that donate hydrogen bonds to the solvating water molecules (green protons in Fig. 4e) lead to progressively redshifted peaks (green spectra in Fig. 4c). Their response approaches the frequency of the bare Eigen cation, yet it originates from asymmetric Zundel-like configurations. This implies a systematic strengthening of the hydrogen bonds within the Eigen-like fragment H_3_O + (H_2_O)_3_ the more asymmetric the overall H_3_O + (H_2_O)_5_ complex becomes. In contrast, the stretching response of the proton that donates the hydrogen bond from H_3_O + (H_2_O)_3_ to the remaining second-shell water molecule (purple proton in Fig. 4e) blueshifts (purple spectra in Fig. 4c), corresponding to a weakening of that hydrogen bond in the very asymmetric H_3_O + (H_2_O)_5_ limit.

## Discussion

In the following, we discuss how the spectroscopic response of eZ, as a hydrogen-bonding solvation tunable model via water detachment, can be mapped to the spectrum of the proton continuum. We first address the featureless band from ~1,000 to 1,400 cm^−1^ in the experimental difference spectra (within the yellow frequency window highlighted in Fig. 2d). Our quantum dynamics simulations suggest that this featureless band is due to the response of the central proton hydrogen bond (yellow window, Fig. 4b) within slightly asymmetric local solvation environments (yellowish curves, Fig. 4d). A careful analysis of the quantum-mechanical cross-correlation between the central proton motion and the *ungerade* bending wavefunctions of the Zundel core (Methods) indicates that the IR response of the central proton hydrogen bond becomes strongly blueshifted in an asymmetric environment due to changes in its local potential energy landscape. As the IR response of the excess proton shifts continuously with asymmetry, the *z* coordinate remains strongly and anharmonically coupled to the asymmetric bending of the inner water molecules, whose IR response remains near 1,750 cm^−1^. In fact, this coupling results in a cross-peak in the 2D IR spectra at excitation and detection frequencies of ~1,200 and 1,750 cm^−1^, respectively^44,47^, and supports assigning the proton continuum band at 1,750 cm^−1^ to mixed proton transfer motion and inner-water bending based on multiple approaches^4,33,36,40^.

In comparison to these findings, classical nuclear dynamics simulations based on a semi-empirical multistate empirical valence bond (MS-EVB) potential combined with a point charge model of the dipole moment to describe the excess proton in bulk water attribute this continuum band to the proton transfer motion^31^. This assignment is not in complete contradiction with our findings and the earlier references, as there is strong coupling between the central proton coordinate (*z*) and the *ungerade* bending of the Zundel subunit for all asymmetric structures considered (see Methods for a wavefunction-based analysis).

In the liquid state, the sharp transition centred between 1,590 and 1,625 cm^−1^, depending on the experiment^34,44,46,47^ (Fig. 2d), previously attributed to ‘a different mode of bending vibration perhaps derived from bulk-like solvation shell water molecules’^47^, is assigned here to the response of the bending modes of the outer, second-solvation-shell water molecules. These appear at 1,620 cm^−1^ in the calculated spectrum of the eZ complex (band a_11_ in Fig. 3a). Inspection of Fig. 3b shows that this sharp peak is unrelated to the response of the asymmetric bending mode of the inner water molecules.

This assignment is further supported by quantum simulations of the IR spectrum of the bare E cation^56^, which report the same assignment for the bending of the three outer water molecules near 1,620 cm^−1^. Thus, the coupling of the aqueous proton to the bending modes of its second solvation shell—meaning either the four external water molecules in Zundel-like structures or the three outer water molecules in Eigen-like structures—accounts for the sharp response at 1,620 cm^−1^ in bulk water. This assignment also supports associating a peak near 1,650 cm^−1^ in the transfer-waiting contribution ‘to a weak coupling to an unperturbed water bending mode of the flanking water molecules’^4^. The fact that the frequency of this second-shell response is conserved across Zundel-like and Eigen-like structures explains its relative narrowness and prominence in the spectral range of the proton continuum.

Next, we discuss the broad spectral features spanning 1,700–1,900 cm^−1^ within the blue frequency window in Fig. 2d. Compared with Fig. 4b, this broad band arises from the combined response of the central proton hydrogen bond in increasingly asymmetric environments and the strongly and anharmonically coupled inner water bending modes. As discussed earlier, the strong coupling of the central proton to the *ungerade* bending modes of the Zundel core results in the doublet structure seen for *R*_const_ > 3 Å in Fig. 4b. Moreover, a comparison of Figs. 2d and 4b shows that the response of the aqueous proton extending towards 2,000 cm^−1^ and up to ~2,300 cm^−1^ also originates from increasing asymmetry involving both the most active proton and Zundel core bending motion. The maximum of this band at 1,750 cm^−1^ is explained by the similar position of the asymmetric inner water bending—both in the Z and eZ complexes and in asymmetric environments—as well as its strong coupling and intensity borrowing from the central proton, regardless of the asymmetry-dependent spectral position of the latter. This response, found in both our asymmetric eZ complexes as well as in the liquid state, extends well into the otherwise silent spectral region of the isolated eZ complex between regions III and IV in Fig. 3.

Interestingly, the green and purple frequency windows in the experimental liquid-state spectra in Fig. 2d are linked to two distinct OH-stretching motions, which shift towards lower and higher frequencies with asymmetry. This implies that the broad feature in the experimental difference spectra in the enormous frequency regime that extends from ~2,600 to ~3,500 cm^−1^ (green and purple frequency windows in Fig. 2d) is a direct signature of the asymmetry of local solvation environments felt by the excess proton in water. This asymmetry leads to both stronger (redshifting) and weaker (blueshifting) hydrogen bonds around the nascent hydronium core (note that the qualitatively different shapes of the four experimental spectra at these frequencies strongly depend on the subtraction procedure).

Finally, the frequency range between ~2,300 and 2,600 cm^−1^ appears as a spectroscopic ‘no man’s land’, as no characteristic modes and thus no IR resonances or peak-like features that directly involve the excess proton in asymmetric eZ structures can be found therein. This strongly suggests that the response in this only mildly modulated and essentially featureless frequency region cannot be explained by asymmetric Zundel-like motifs in the condensed phase. In fact, pulling two water molecules from a perfect Eigen environment by a few tenths of an angstrom suffices to bring the response of the shortest hydrogen bond from 2,700 cm^−1^ down into the aforementioned frequency range^56^, thus supporting the idea that this intermediate spectral region may be dominated by an ensemble of statistically less abundant Eigen-like structures.

## Conclusions and outlook

Our full-dimensional quantum dynamics approach provides a stroboscopic perspective of the IR response of the excess proton. This is achieved within the framework of the eZ complex, H^+^(H_2_O)_6_, as an accurate spectroscopic model that mimics key modulations of the potential energy landscape for proton transfer due to hydrogen-bonding fluctuations in local solvation environments. By probing localized vibrational excitations of unperturbed eZ, we were able to assign the most relevant peaks in the IR spectrum and reveal the underlying couplings responsible for the observed spectral features. In particular, we explain the absence of the characteristic Fermi doublet of the Zundel core in eZ. We find that this doublet is suppressed by the local solvation environment around the Zundel core. The outer solvating water molecules are shown to hinder the wagging modes of the inner Zundel core, resulting in an intense and unstructured band corresponding to the central proton hydrogen bond.

In an effort to connect accurate gas-phase quantum dynamics to liquid-phase experiments, we gradually removed a second-shell water molecule to introduce asymmetry and study the continuous transition from symmetric to asymmetric hydrogen-bonding motifs around the transferring proton. In particular, we worked out how the intensity modulations of the broad continuum band emerge due to local solvation asymmetries impacting the quantum dynamics of the excess proton in eZ. Importantly, we highlight the strong coupling of the central proton hydrogen bond and Zundel core bending modes in asymmetric solvation environments as the origin of the aqueous proton’s response in the 1,700–2,300 cm^−1^ spectral region; tracing the position of its maximum at 1,750 cm^−1^ to the dominance of the Zundel asymmetric bending in shaping this spectral response. In contrast, the bending mode of second-shell waters conserved across Zundel-like and Eigen-like structures is responsible for the narrower band of the proton continuum centred at 1,620 cm^−1^.

Having demonstrated the performance of our approach, it would be highly interesting to address next the broad continuum IR absorption of hydroxide in water, that is, alkaline aqueous solutions. Given the much more complex local hydrogen-bonding motifs of OH^−^(aq) subject to Grotthuss diffusion—particularly the interplay of hypercoordination in the resting state and tetrahedral hydrogen bonding in the active state—another methodological leap in computational spectroscopy is required to handle the increased dimensional complexity. Thus, our progress on high-dimensional quantum dynamics simulations opens future research avenues in fundamental acid–base chemistry.

## Methods

### Spectroscopic model of proton micro-solvation based on the eZ complex

The asymmetric Zundel complex is currently discussed as the most relevant structure for the aqueous proton according to the latest 2D IR experiments^44,46,47^. Furthermore, the definition of ‘Zundel-like’ complexes has been broadened such that it also encompasses ‘configurations that might be best characterized geometrically as more Eigen-like’^47^, and very recent path integral molecular dynamics simulations based on density functional theory have identified a novel doubly-gated proton transfer mechanism characterized by sequential hydrogen-bond exchanges and a long residence time in Zundel-like environments^39^. In this context, the eZ motif (H_13_O_6_^+^) provides an ideal infrared spectroscopic model for linking Zundel- and Eigen-like micro-solvation structures to local bulk solvation environments, as it is the smallest cluster that has both structures completely embedded in it. Moreover, a pronounced similarity in the IR spectrum of eZ and the larger protonated water clusters H^+^(H_2_O)_7_ and H^+^(H_2_O)_8_ has been reported^11^; the latter display only a few additional sharp bands corresponding to different types of dangling O–H stretching mode. This further supports the use of the eZ complex as a valid infrared spectroscopic model, without the need for introducing an additional hydrogen-bond-donor water molecule.

To accomplish this, we first rigorously compute the IR spectrum of the eZ complex in the gas phase, using chemically relevant curvilinear coordinates in full dimensionality (51 vibrational coordinates) and in conjunction with highly accurate, first-principles potential energy and dipole moment surfaces. The resulting IR spectrum is in excellent agreement with gas-phase experiments without introducing any adjustable parameters, thereby validating the overall approach. We note that, up to now, no other combination of quantum dynamics, electronic structure methodology and potential energy fitting technology reproduces the full IR spectral range of such complex species with this reported accuracy. Following this, we use eZ as a template to generate a continuum of structures, from fully symmetric to strongly asymmetric, that aim to mimic different micro-solvation environments while including second-shell effects.

### Quantum molecular dynamics simulations

We employ the multilayer multiconfiguration time-dependent Hartree (ML-MCTDH) method^62–64^, a rigorous quantum dynamics methodology offering several key advantages to accurately compute the IR response of the hydrated proton. Being a wavefunction approach, ML-MCTDH does not impose onto molecular motion the quasi-classical approximations that underlie all practical path integral-based quantum dynamics methods used in condensed phase simulations^65,66^. Due to its time-dependent, variational nature and highly compact wavefunction representation, it also transcends the restrictions of vibrational mean field theory (VSCF)-based methods such as VPT2 or VCI when applied to finite protonated clusters: it captures most correlations while computing the whole spectrum using the correlation function of one or few propagated wavepackets. Therefore, the ML-MCTDH approach allows us to tackle proton transfer dynamics, including quantum effects and polarization fluctuations derived from the different local solvation environments encompassed within eZ (Fig. 1b), without applying dimensionality reduction or fragment approximations. This is made possible thanks to recent technical developments for the optimal tensor network decomposition of correlated wavefunctions^67–69^, in conjunction with a compact representation of the interaction potential^70,71^ and the implementation of dedicated software that provides analytic expressions of the kinetic energy operator^72–74^.

### Coordinate system for nuclear motion

When performing quantum dynamic simulations, a key issue is selecting the appropriate set of coordinates to describe the molecular process. In the present work we employ a set of polyspherical curvilinear coordinates, that is, coordinates involving distances and angles, which have been found crucial for the description of the large amplitude motions and anharmonicities in this and other protonated water clusters^56,57^. The full set is extensively described in Supplementary Section 2, so here we focus on a subset of the more relevant motions to understand the main results presented in the Article. As depicted within a scheme of the eZ complex in Extended Data Fig. 1a, the first set, coloured in orange, includes the central proton coordinate (*z*), which describes the corresponding proton transfer motion, and two other coordinates, the inner-layer oxygen–oxygen distance (*r*) and wagging motion (*γ*). The second set in green contains the two analogous outer-layer motions (*R* and *Γ*), as well as the two libration or orientational angles (*Θ* and *Θ*′) of the local solvation environment.

### Potential energy surface

Rather than using density functional theory as in most practical condensed phase simulations of IR spectra, the present simulations rely on our full-dimensional potential energy and dipole moment surfaces^75–77^, which are based on very accurate neural network representations of CCSD(T)-grade coupled cluster data^78^; excellent test errors of 0.06 kJ mol^−1^ per atom and 0.03 D were achieved for the potential energy and dipole moment surfaces, respectively. We explicitly tested the performance of the neural network potential energy representation against the coupled cluster reference for the potential energy profile along the central transfer coordinate *z* for three representative cases of the asymmetric removal of one water molecule, shown in Fig. 4d. As shown in Extended Data Fig. 2, we find very good agreement overall of the potential energy profiles across the entire range of *z* for all three cases, further highlighting the ability of the neural network potential to give a faithful description of the central proton mode in all cases of asymmetry. See Supplementary Section 3 for more details.

### Correlation analysis of the many-particle nuclear density distribution

In highly anharmonic systems featuring multiple minima, such as in the case of eZ, the correlation of the quantum-mechanical wavefunction becomes too large to handle, as excitations along many rectilinear normal modes must be involved simultaneously to account for the naturally curvilinear displacements of the system. This is illustrated in Extended Data Fig. 1b, where the correlations derived from our potential energy surface of eZ are arranged as a matrix, in which the values for each pair of degrees of freedom quantify the strength of their coupling. For instance, the first three coordinates in the top-left corner form a sub-block associated with the Zundel core (orange square), while the remaining four vibrational degrees of freedom form a larger lower sub-block belonging to the local solvation environment (green square). These two blocks are in turn coupled through the respective off-diagonal elements (blue rectangle), revealing the underlying complex interactions that give rise to the IR spectra discussed in Supplementary Section 2A. In particular, some notable patterns emerge; in the first place, certain solvation environment coordinates exhibit substantial coupling among themselves, while remaining largely decoupled from the Zundel core; this is the case for coupling between the outer wagging (*Γ*) and the libration angles (*Θ* and *Θ*′).

In contrast, other coordinates not only display strong correlations within their respective diagonal sub-block (orange and green squares), but also present pronounced correlations in the off-diagonal (blue rectangle) block. In particular, the three main coordinates associated with the Fermi resonance of the bare Zundel (*z*, *r*, *γ*), and the four outer oxygen–oxygen distances (*R*) of the second solvating shell, are strongly coupled. The emerging mechanistic picture is that the four solvating water molecules in the eZ complex hinder the dynamics of the inner Zundel core, especially the wagging motion (*γ*), which is key to the mechanism of the Fermi resonance in the bare Zundel cation. On the other hand, no strong disturbance of the bare Zundel bending motion by the outer water molecules was found in the correlation analysis of eZ. For a complete analysis in full dimensionality and the details of the numerical procedure, which was adapted from ref. ^69^, we refer to Supplementary Section 3.

### IR spectrum of the eZ complex in equilibrium

The calculated spectrum was obtained via Fourier transform of the dipole–dipole correlation function of duration 2 ps, hence having a resolution of ~15 cm^−1^. The calculations are based on a fully quantum 51D treatment of the vibrational wavefunction that provides excellent agreement with the experimental spectrum. In particular, the accuracy of all calculated bands lies within the spectral resolution when compared with experiment, and we note that no global frequency shift has been applied to the calculation. This indicates a tightly converged vibrational wavefunction propagation and highly accurate potential energy and dipole moment surfaces. Technical details and assignments are described in Supplementary Section 7.

### Desolvation of eZ via water detachment

We define the parameter *R*_const_ as the distance between two oxygen atoms. One of the oxygen atoms belongs to an inner water of the Zundel core, while the other oxygen atom belongs to one of the four outer water molecules (Fig. 4e). We then systematically distort this spectroscopic model in a twofold manner: (1) by gradual removal of all four solvating water molecules in a perfectly symmetric way in order to disclose the impact of local solvation effects on the characteristic Fermi splitting of the bare Z motif and (2) by pulling away only one of the outer-layer solvation water molecules in quasi-continuous steps. In other words, *R*_const_ now becomes a continuous asymmetry parameter that seamlessly transforms Zundel-like into Eigen-like eZ complexes upon increasing its magnitude beyond the equilibrium value, thereby systematically probing the impact of local solvation asymmetry around the excess proton^35^ on the total IR response within a micro-solvation environment. We refer to Supplementary Section 8 for more details about the implementation.

### Cross-correlation analysis

Analysis of the quantum-mechanical cross-correlation between the central proton motion (*ψ*_*z*_) and *ungerade* bending (${\psi }_{{\mathsf{u}}-{\mathsf{bend}}}$) wavefunctions of the Zundel core reveals that the doublet in the spectrum of asymmetric structures in Fig. 4b corresponds to hybridized eigenstates ${\phi }_{\pm }=a\,{\psi }_{z}\pm b\,{\psi }_{{\mathsf{u}}-{\mathsf{bend}}}$ in terms of zeroth-order excitations. The frequency difference between the two peaks of the doublet is ~400 cm^−1^ for *R*_const_ ≈ 3.9 Å, indicating a coupling strength of half this value in an idealized, two-state model, and close to the estimate of 260 cm^−1^ for this coupling in the bare Zundel cation^54^. This joint response of the *ψ*_*z*_ and ${\psi }_{{\mathsf{u}}-{\mathsf{bend}}}$ excitations forming a doublet, however, cannot be recovered by linear absorption spectroscopy due to the continuous fluctuations of the solvation environment. It is, however, clearly seen when examining the cross-peaks in 2D-IR measurements^47^ at excitation frequency 1,200 cm^−1^ and detection frequency 1,750 cm^−1^. When considering all this evidence together, we conclude that the central proton coordinate and *ungerade* bending modes of the Zundel subunit in eZ are strongly mixed. See Supplementary Section 9 for a quantitative analysis.

## Online content

Any methods, additional references, Nature Portfolio reporting summaries, source data, extended data, supplementary information, acknowledgements, peer review information; details of author contributions and competing interests; and statements of data and code availability are available at https://doi.org/10.1038/s41557-026-02209-3.

## Supplementary information

Supplementary Information Supplementary discussion sections 1–10, Figs. 1–12 and Tables 1–3.

## Data Availability

Source data files for all data presented in graphs within the figures and Extended Data are provided, together with all the necessary input files compatible with the Heidelberg MCTDH package. These data are accessible from https://doi.org/10.5281/zenodo.20411426 (ref. ^79^).

## Code availability

The TANA and MCTDH codes, together with their full documentation and any further input files needed to reproduce particular results of the current contribution, are available upon request from the authors.
