# Supplementary material for: Deciphering the infrared spectrum of the hydrated proton using full-dimensional quantum dynamics
Source: Nat Chem. 2026 Jul 27;18(10):1773–80. doi: 10.1038/s41557-026-02209-3 (PMC13623638; doi:10.1038/s41557-026-02209-3)
Supplement: Supplementary file 1 [file 41557_2026_2209_MOESM1_ESM.pdf]

nature chemistry

Supplementary information

https://doi.org/10.1038/s41557-026-02209-3

Deciphering the infrared spectrum of the hydrated proton using full-dimensional quantum dynamics

In the format provided by the authors and unedited

CONTENTS

Overview 1

S1. Extended discussion of previous work 1

S2. Coordinate system and kinetic energy operator 3

S3. Neural network potential and dipole moment surfaces, sampling points and analysis of correlations 5

S4. Wavefunction representation and primitive basis set 9

S5. Potential energy and dipole moment surface fits 9

S6. N-mode representation of dipole surfaces 10

S7. Assignment of the IR spectrum of the extended Zundel in the gas phase 12

S8. Potential energy cuts and reduced dimensionality calculations 13

S9. Cross-correlation analysis between localized excitations: Unraveling the proton transfer and inner-water bending coupling 14

S10. Counterion impact in the liquid state 16

References 18

OVERVIEW

Gas-phase infrared (IR) spectroscopy of protonated water clusters of the form $H^+(H_2O)_n$ has become an important tool to understand the nature of a proton embedded in water, i.e., H^+(aq). In this regard, here we perform full-dimensional quantum dynamics simulations of the extended Zundel complex (eZ: ${H_{13}O_6}^+$), which structurally encompasses both so-called Zundel (Z: ${H_5O_2}^+$)[1] and Eigen (E: ${H_9O_4}^+$)[2] archetypes. To be more precise, Fig. S1(a) illustrates ${H_{13}O_6}^+$ as a Zundel subunit (orange) solvated by four water molecules, while Fig. S1(b) shows it instead as an Eigen subunit (green) bound to two solvating water molecules. In the following sections, we detail the technical aspects of these computations by focusing on ten main facets:

1. Extended discussion of previous work
2. Coordinate system and kinetic energy operator
3. Neural network potential and dipole moment surfaces, sampling points and analysis of correlations
4. Wavefunction representation and primitive basis set
5. Potential energy and dipole moment surface fits
6. N-mode representation of the dipole surfaces
7. Assignments of the IR spectra in the gas phase
8. Potential energy cuts and reduced dimensionality calculations
9. Cross correlation analysis between localized excitations
10. Counterion impact in the liquid state

Throughout, we also distinguish each of the six water molecules in $H^+(H_2O)_6$ by Roman numerals (I-VI) as illustrated in Fig. S1(c).

S1. EXTENDED DISCUSSION OF PREVIOUS WORK

This section provides an extended discussion of previous computational and experimental work on the IR spectrum of the hydrated proton complementing the main text.

Given the complexity of the aqueous proton, it is no surprise that a wealth of ideas has been advocated to explain the proton continuum band in the computational literature; sometimes published jointly with experimental studies. These include the concept of a dynamically distorted Eigen cation with relatively little contribution from traditional limiting Zundel configurations [3], a "clusters-in-liquid" approach assigning a peak near 1740 cm^{-1} to the proton-transfer mode in acidic aqueous solutions [4], or a combination of local Eigen-like and Zundel-like configurations contributing throughout the entire infrared spectrum [5]. Other explanations include classical Eigen-like and Zundel-like structures giving rise to similar spectra subject only to intensity differences [6], demonstrations of how the asymmetry in the proton's solvation environment impacts the vibrational density of states, highlighting the significance of a broad range of proton-sharing environments [7], decompositions of the full IR spectrum uncovering distinct spectral contributions as proton configurations transition from more Zundel-like to more Eigen-like motifs [8], indications that fluctuations between Eigen-like, intermediate, and Zundel-like configurations might best describe experimental 2D IR spectra [9], and anisotropy reorientation time-scale analyses that do not lend support to a distorted Zundel cation picture [10] as suggested earlier by nonlinear spectroscopy [11, 12].

The interpretation of the condensed phase spectral response is further complicated by the role of counteranions, also discussed later in Sec. S10. Compared to 2D-IR liquid phase spectroscopy, typically requiring 2–4 M acid concentrations, gas phase experiments are free of any counterion effects, thus providing a powerful independent avenue to complement such liquid-state studies. Finite protonated water clusters [13] have again been studied both with experimental [14–24] and computational [4, 5, 8, 9, 25–32] means. Hence, the key idea behind the cluster approach toward "demystifying the diffuse vibrational spectrum of the aqueous proton through cold cluster spectroscopy" [13] has been to probe protonated water clusters of the form $H^+(H_2O)_n$ [14, 15, 18]. To be more precise, the messenger species X used in vibrational predissociation techniques is deliberately deployed as a non-innocent tag, intended to distort the equilibrium structure of the resulting $H^+(H_2O)_n{\cdot}X_m$ heteroclusters [21, 23, 27]. Using tags such as $X = CH_4$, D_2, N_2 or CO including D_2O perturbs the potential energy landscape of the excess proton, thereby driving the H-bonding

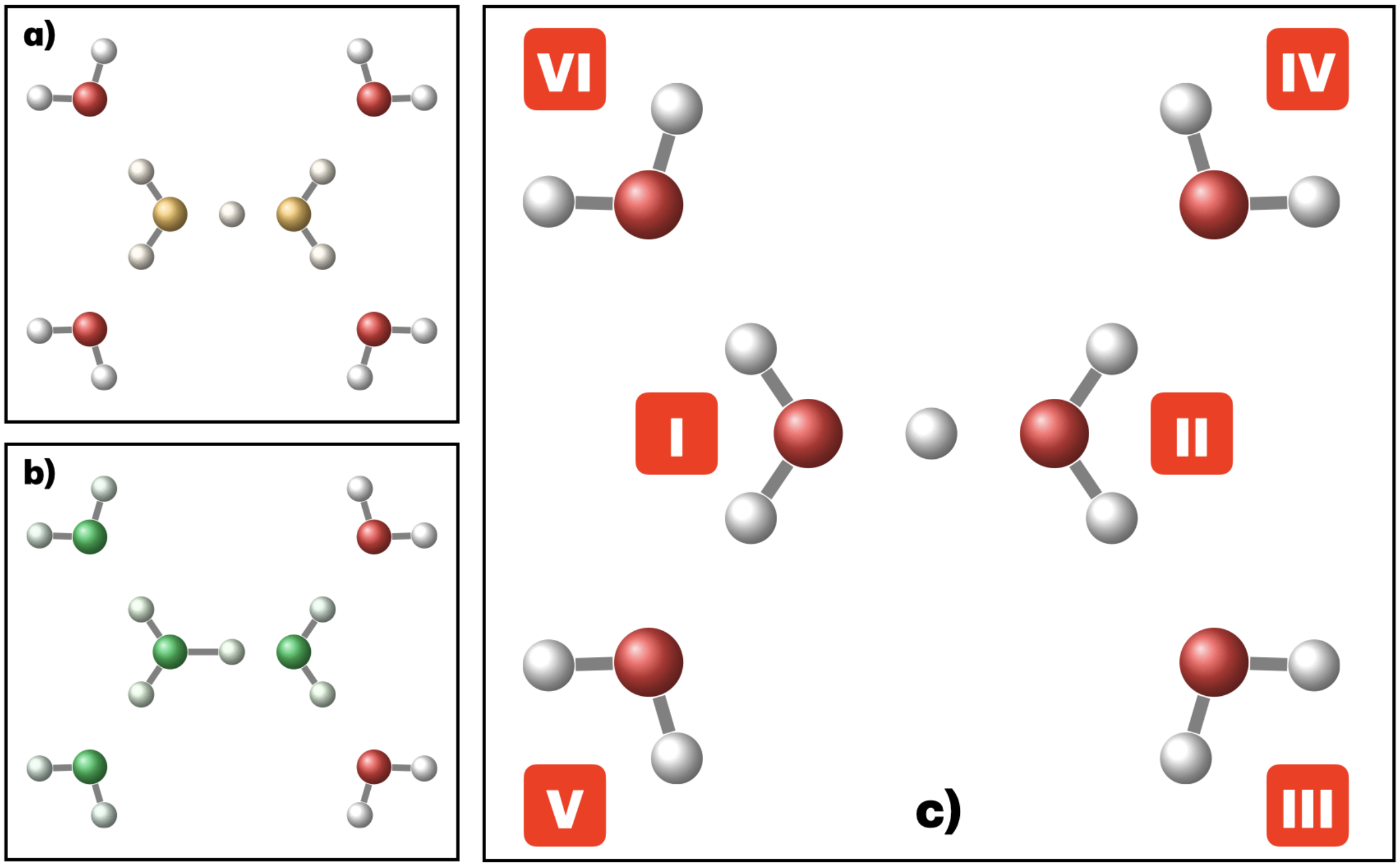

FIG. S1. Multifaceted structure of the extended Zundel complex: a) $H_{13}O_6{}^+$ seen as a Zundel-core surrounded by a first solvation shell, i.e., $H^+(H_2O)_2(H_2O)_4$, b) $H_{13}O_6{}^+$ seen as a Eigen cation bound to two water molecules, i.e., $H^+(H_2O)_4(H_2O)_2$, and c) labeling of the water molecules surrounding the central proton in $H^+(H_2O)_6$.

environments from Zundel-like to Eigen-like motifs in discrete steps [21, 23, 27]. Notably, the IR response of the excess proton in such different environments has been traced through suitable manipulations of coordinating water molecules of the $H^+(H_2O)_6$ parent complex [23]. This culminated in previously unseen insights into the assignment of IR resonances, as they evolve through a few snapshots along a coarse-grained proton transfer pathway [13]. Still, a limitation shared by all flavors of this technique is the fact that the structure of such finite hydrated proton models can only be changed in a stepwise manner by modifying the chemical composition of $H^+(H_2O)_n{\cdot}X_m$. Evidently, this differs significantly from the situation encountered in the liquid phase, where, in the absence of X, H-bond and polarization fluctuations generate a continuous range of local solvation environments [31], thereby directly influencing the IR response of the dynamically evolving excess proton as it migrates through Grotthuss diffusion.

As one can see, despite many studies on the topic, this long-standing debate regarding the nature of the aqueous proton remains open. In this regard, a new twist to the story was recently added with the proposal of a novel three-step proton transport mechanism to explain Grotthuss diffusion and consequently, the continuum IR absorption of the aqueous proton [33]. This idea is based on a thermal equilibrium involving two stable proton-localized structures with distinct Eigen-like and Zundel-like H-bond motifs. Notably, in the stable "Zundel-like H-bond motif", the excess proton is localized closer to one of the two inner water molecules, yet within a larger symmetric H-bonding environment [33]. In other words, once again we find the recurrent key role of an "asymmetric Zundel-like $H_5O_2^+$" motif. In this case, the transferring proton within the most active H-bond [34] — also dubbed special bond [35] or special pair [36] — forms a hydronium core H_3O^+, whilst the "ideal Zundel structure" with a centered H-bond is found to be slightly unstable [33].

Nonetheless, it remains unclear how these species contribute to the observed spectral features in the aqueous proton, particularly how the local environment and asymmetry affect the IR response in the solvated $H_2O\cdots H^+\cdots OH_2$ motif. This is the question we address in the present work through a "stroboscopic movie" approach based on full-dimensional quantum dynamics of the extended Zundel complex, as detailed in the main text.

S2. COORDINATE SYSTEM AND KINETIC ENERGY OPERATOR

When performing quantum dynamic simulations, a key issue is selecting the appropriate set of coordinates to describe the molecular process. For example, a simple harmonic model can effectively describe molecular vibrations for small-amplitude motions around a given equilibrium geometry in rigid systems. This is because, in this case, the well-known rectilinear normal coordinates render the Hamiltonian operator nearly separable around the reference configuration. However, in highly anharmonic systems, possibly featuring multiple minima — such as in the case of the extended Zundel complex ($H_{13}O_6{}^+$) — normal coordinates fail to describe molecular motions naturally: The correlation of the quantum mechanical wavefunction (which is not an observable physical quantity but a mathematical measure of a function that depends on the chosen coordinate set) becomes too large to handle as excitations along many rectilinear normal modes must be involved simultaneously to account for the naturally curvilinear displacements of the system. We call this phenomenon "artificial correlation", and it can be countered by a thoughtful selection of curvilinear coordinates to describe the vibrational dynamics.

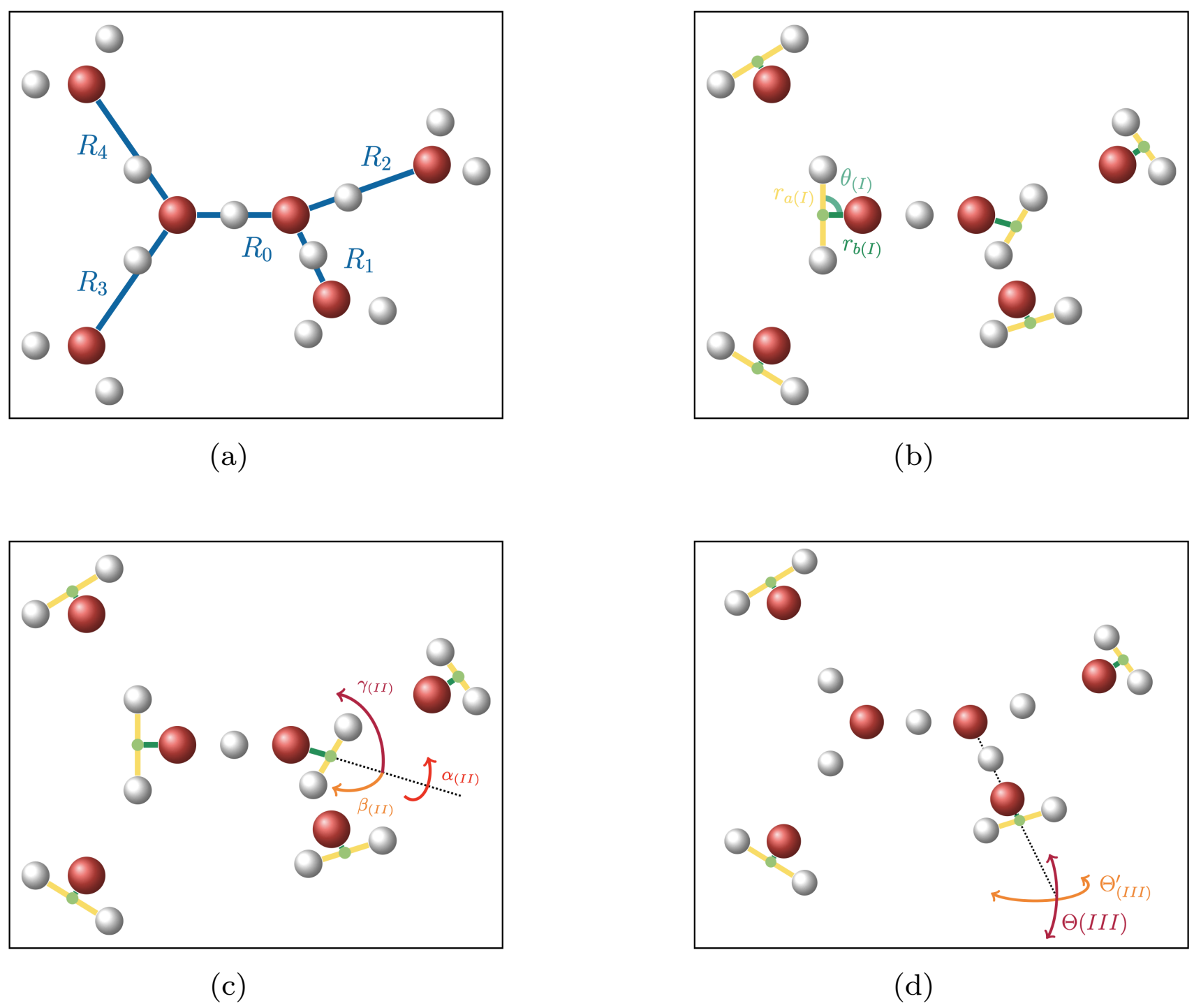

FIG. S2. Sets of polyspherical coordinates used in the extended Zundel cation.

In the present work we employ a set of polyspherical curvilinear coordinates, i.e., coordinates involving distances and angles, which have been found crucial for the description of the large amplitude motions and anharmonicities in this and other protonated water clusters[25, 31]. These are further illustrated in Fig. S2 as follows:

(a) 5 oxygen-oxygen vectorial distances ($\vec{R}_0$, $\vec{R}_1$, $\vec{R}_2$, $\vec{R}_3$ and $\vec{R}_4$).

(b) 18 so-called Jacobi coordinates; one set ($\vec{r}_a$, $\vec{r}_b$ and θ) for every water molecule (6×3).

For each water molecule, $\vec{r}_a$ is the vectorial distance between the hydrogen atoms, $\vec{r}_b$ is the vectorial distance between the oxygen atom and the center of mass of the H_2 fragment, and θ is the angle between these two vectors.

(c) 17 hydrogen orientation ("Euler") angles; one set (α, β, and γ) for every water molecule, except for the first defined unit, which acts as an anchor ($6 \times 3 - 1$).

These angles define the three relative orientations of the OH_2 fragments as torsion (α), rocking (β) and wagging (γ).

(d) 8 "libration" angles corresponding to the spherical coordinates associated with the four "valence" oxygen-oxygen distance vectors $\vec{R}_0$, $\vec{R}_1$, $\vec{R}_2$, $\vec{R}_3$ and $\vec{R}_4$. This results in one coordinate pair (Θ and Θ') for every vector shown in (a).

(e) 3 Cartesian coordinates (x, y and z) describing the central proton.

which makes a total of 51 degrees of freedom in full dimensionality, i.e., $3N - 6 = 51$ since the number of atoms is $N = 19$.

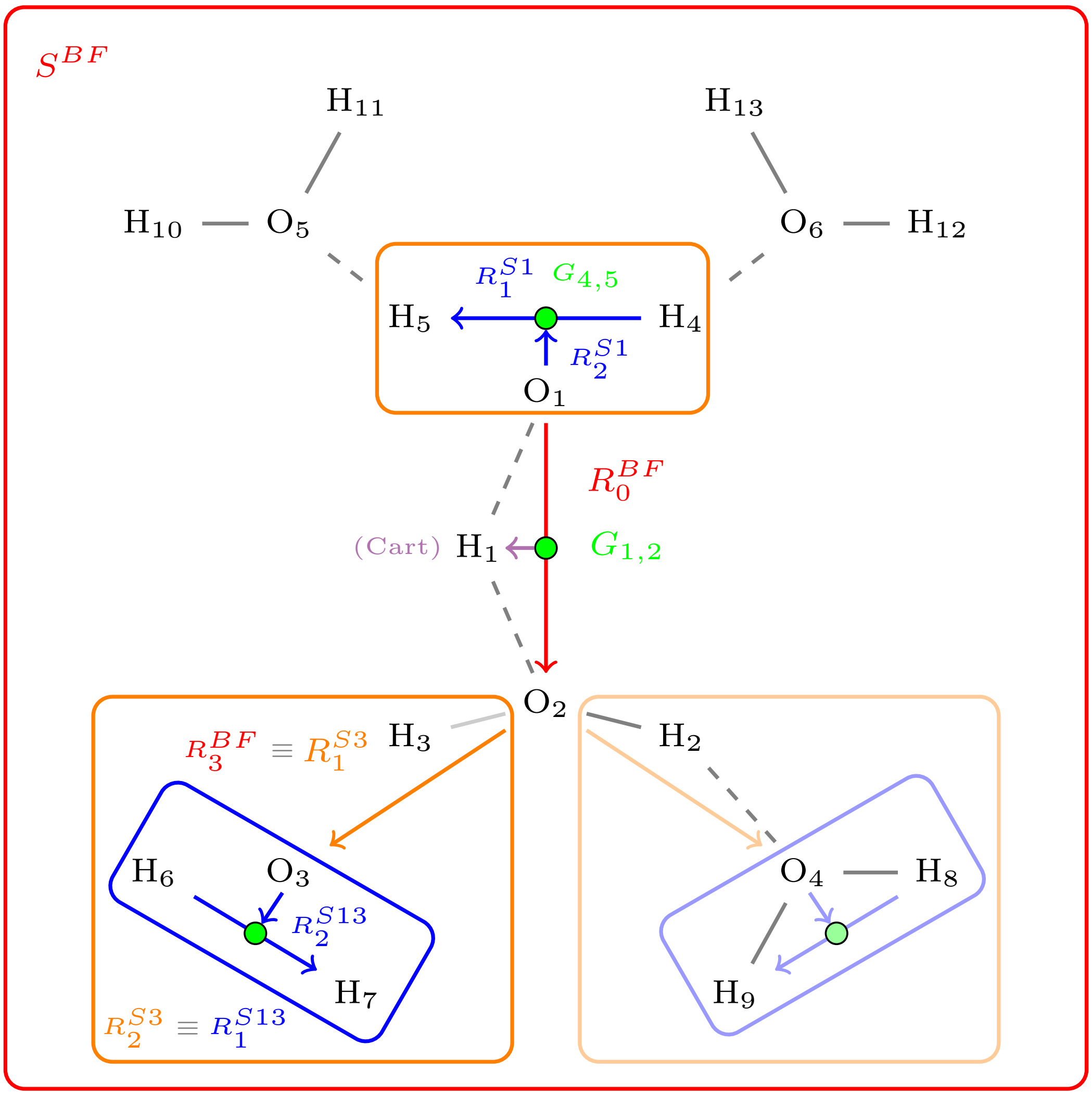

FIG. S3. Structure of the subsystem vectors for the description of extended Zundel complex in polyspherical coordinates. The resulting KEO contains a total of 8488 terms.

The so-called polyspherical approach, which has been already presented in a series of papers [37–48], provides a general formulation to obtain the exact kinetic energy operator (KEO) of a given N-atom system employing curvilinear coordinates for a specific family of spherical vectors. In addition to their great flexibility in terms of coordinate choices (e.g., valence, Radau, Jacobi, Cartesian and combinations thereof), these coordinates always yield a KEO in a sum-of-products form composed of one-dimensional terms, which is essential for our MCTDH implementation[49].

In the standard polyspherical coordinate framework, the polyspherical vectors — whether valence, Radau, Jacobi, or combinations thereof — are parameterized using spherical coordinates defined with respect to the first two vectors, which together establish the body-fixed frame of the molecule. For large systems, such as the extended Zundel complex, this standard formulation can introduce artificial couplings between motions that are physically decoupled. For example, between atoms located in distant regions of the molecule. This is the motivation behind generalizing the polyspherical approach by grouping atoms into subsystems. Each subsystem is assigned its own local body-fixed frame, and the vectors within the subsystem are defined locally with respect to this frame. This approach allows for

the decoupling of motions between atoms belonging to different partitions, thereby improving the physical accuracy of the representation.

In particular, Fig. S3 illustrates the subsystems employed for the eZ complex, where $\vec{R}_0^{\rm BF}$ (red) represents the valence vector connecting the two central oxygen atoms, O_1 and O_2. Due to the molecule's symmetry, this is the natural choice to define the body-fixed (BF) frame. The BF (x, z), with the half-plane $x > 0$, is chosen so that the valence vector connecting O_2 and O_3, i.e., $\vec{R}_3^{\rm BF}$ (red), lies within this half-plane. Two subsystems are then defined, one for each of the inner water molecules of the Zundel-core, with the first shown as an orange rectangle at the upper side of Fig. S3. The corresponding Jacobi vectors for the atoms O_1, H_4 and H_5 are also depicted as $\vec{R}_1^{S1}$ and $\vec{R}_2^{S1}$ (blue), where $G_{4,5}$ is the center of mass between H_4 and H_5. The same vectors are defined for the second subsystem (i.e., $\vec{R}_1^{S2}$ and $\vec{R}_2^{S2}$ for O_2, H_2 and H_3), but they are not drawn for the sake of simplicity, as they have the same definitions. This "separation into two subsystems" is described in Ref. [42] (see Eq. (37) there), and that this is the same choice of vectors employed in previous studies of the bare Zundel cation[50–56].

Following this, we describe the remaining four subsystems. Two of them are depicted by the orange rectangles at the bottom of the figure. The corresponding (local) z-BF axes are parallel to the valence vectors joining O_2 and O_3 ($\vec{R}_1^{S3}$, orange) or O_2 and O_4. The same applies to the top of the figure for the two vectors joining O_1 and O_5 or O_1 and O_6, although they are not shown for simplicity. Once again, the local BF (x, z), with the $x > 0$ half-plane, is defined such that the Jacobi vector joining the hydrogen atoms in the outer water molecules is parallel to it (e.g., the vector joining H_6 and H_7, $\vec{R}_2^{S3}$, in orange). Then four "sub-subsystems" are defined, one in each of the aforementioned subsystems, meaning that there is one for each of the outer water molecules. These are illustrated in the lower part of the figure. As before, we do not draw the vectors for all of them, since the definitions are equivalent; thus only the Jacobi vectors $\vec{R}_1^{S13}$ and $\vec{R}_2^{S13}$ (blue) are shown as representative examples. Finally, the central proton H_1 is described using Cartesian coordinates, with the origin at the center of mass between O_1 and O_2, denoted as $G_{1,2}$.

Analytic expressions for the KEO and the corresponding back-and-forth transformations — i.e., between the Cartesian coordinates of the atoms and the internal coordinates of the molecule — were derived using the TANA software developed by David Lauvergnat[57, 58]. For further details regarding the implementation, see Refs. [59–63], while the generalized separation into subsystems technique is described in Section 2.4 of Ref. [37].

S3. NEURAL NETWORK POTENTIAL AND DIPOLE MOMENT SURFACES, SAMPLING POINTS AND ANALYSIS OF CORRELATIONS

The values of the potential energy surface (PES) and dipole moment surface (DMS) at individual geometries were obtained from a neural network (NN) model trained on highly accurate coupled cluster reference data expressed in Cartesian coordinates. The development, detailed benchmarking and validation of both are described in Refs. [64-65] and [66], respectively; these references demonstrate the excellent performance of the two NN models in reproducing the reference potential energy and dipole vector data and derived observables. Accordingly, in what follows we provide only a brief overview of the NN models employed here.

Both NN-PES and NN-DMS were trained on protonated water clusters of increasing size, i.e., $H^+(H_2O)n$ with n=1-4, while also including single water molecule configurations. Energies were obtained at the essentially basis-set-converged CCSD(T*)-F12a/aug-cc-pVTZ level, while dipole moments were computed at the DF-CCSD(T*)-F12a/aug-cc-pVDZ level with negligible basis set errors relative to aug-cc-pVTZ. Excellent test errors of 0.06 kJ/mol per atom and 0.03 D were achieved for the PES and DMS, respectively. Application to the eZ complex was shown to be similarly accurate, yielding test errors between 0.11-0.16 kJ/mol per atom [65] and 0.05 D [66], thus demonstrating the capability of these NN models to accurately describe the potential energy and dipole moment landscapes of such a large system.

In order to obtain accurate quantum configurational distributions on that PES, path integral molecular dynamics (PIMD) simulations were carried out with CP2K[67, 68]; the initial configurations corresponded to the CCSD(T*) minimum of the eZ complex $H_5O_2^+(H_2O)_4$. Nuclear quantum effects were treated using the path integral quantum thermal bath (PIQTB) thermostat technique[69] to enhance Trotter convergence, including its ultralow temperature extension[70] that we implemented earlier into CP2K. The (Trotter) number of beads P was chosen according to our previously established convergence criteria when using PIQTB. For every temperature spanning the range from 300 down to 1.67 K (using $P = 6$ and 256, respectively), four statistically independent replicas were propagated for an aggregated simulation time of 1 ns with a 0.25 fs time step; the first 10 ps of each run were discarded as equilibration. The quantum trajectories remained stable across the entire 1.67–300 K range and sampled exclusively the extended Zundel minimum below 100 K. Full details of these simulations and additional analysis of the performance of the NN-PES are discussed in Ref. [65].

These sampling distributions were used to compute correlation matrices following the protocol established in Ref.

[71]. Briefly, this procedure is based on the weighted distance correlation, $dCor$, as defined earlier [72], which measures both linear and non-linear correlations between two variables x and y. Similar to the Pearson correlation coefficient, the idea is to assess how the corresponding sets of vectors $\boldsymbol{x} = [x_1, x_2, \cdots, x_n]^T$ and $\boldsymbol{y} = [y_1, y_2, \cdots, y_n]^T$ tend to covary; however, rather than measuring this in terms of the distances from their respective means, $dCor$ assesses how they tend to covary in terms of distances from all other points:

$$dCor(\boldsymbol{x}, \boldsymbol{y}) = \frac{dCov(\boldsymbol{x}, \boldsymbol{y})}{\sqrt{dCov(\boldsymbol{x}, \boldsymbol{x})}\sqrt{dCov(\boldsymbol{y}, \boldsymbol{y})}} \tag{1}$$

where

$$dCov^2(\boldsymbol{x}, \boldsymbol{y}) = \sum_{j}^{n}\sum_{l}^{n} \hat{X}_{jl}\hat{Y}_{jl} \tag{2}$$

with $\hat{X}_{jl}$ and $\hat{Y}_{jl}$ being the double-centered matrices

$$\hat{X}_{jl} = X_{jl} - \langle X \rangle_j - \langle X \rangle_l + \sum_{j'}^{n}\sum_{l'}^{n} X_{j'l'} \tag{3}$$

$$\hat{Y}_{jl} = Y_{jl} - \langle Y \rangle_j - \langle Y \rangle_l + \sum_{j'}^{n}\sum_{l'}^{n} Y_{j'l'} \tag{4}$$

where $\langle X \rangle_j$ is the weighted mean over the rows, $\langle X \rangle_l$ is the weighted mean over the columns and

$$X_{jl} = \|x_j - x_l\| \tag{5}$$

$$Y_{jl} = \|y_j - y_l\| \tag{6}$$

$$\tag{7}$$

In particular, Fig. S4 illustrates the correlation matrix obtained at a temperature of 1 K, in which two main sets can be distinguished due to the convenient ordering: The 15 variables in the bottom-right corner form a sub-block belonging to the Zundel-core, while the remaining variables form the larger upper sub-block composed by the solvation environment. These two sub-blocks, in turn, are coupled through the respective off-diagonal elements, revealing the underlying complex interrelations.

Therefore, in order to obtain smaller yet representative correlation matrices, the values of groups of coordinates were averaged by combining strongly coupled sub-blocks. These intermediate results, which ultimately led to the correlation matrix analyzed in the main text, are shown in Fig. S5 as:

a) The strongly coupled inner torsion $\alpha_{(II)}$ and the libration angles $\Theta_{(III)}$, $\Theta_{(IV)}$, $\Theta_{(V)}$, $\Theta_{(VI)}$ we combined. Similarly, the other set of librational motions $\Theta'_{(III)}$, $\Theta'_{(IV)}$, $\Theta'_{(V)}$, $\Theta'_{(VI)}$ were also grouped. These two collective motions are labelled as $\boldsymbol{\Theta}$ and $\boldsymbol{\Theta'}$.

b) By joining in one term the labels III, IV, V and VI, the outer Jacobi coordinates were combined into three groups named $\boldsymbol{r}_a$, $\boldsymbol{r}_b$ and $\boldsymbol{\theta}$.

c) The same compilation as before (labels III, IV, V and VI) was applied to the outer torsions, rocking and wagging leading to the new variables $\boldsymbol{A}$, $\boldsymbol{B}$ and $\boldsymbol{\Gamma}$, respectively. Similarly, the outer oxygen-oxygen distances R_1, R_2, R_3 and R_4 were merged into $\boldsymbol{R}$.

d) By joining in one term the labels I and II, the inner rocking, wagging and Jacobi angles were grouped into $\boldsymbol{\beta}$, $\boldsymbol{\gamma}$ and $\boldsymbol{\xi}$, respectively.

Finally, the outer rotation angles $\boldsymbol{A}$, $\boldsymbol{B}$ were merged into $\boldsymbol{AB}$. In addition, the outer Jacobi distances $\boldsymbol{r}_a$ and $\boldsymbol{r}_b$ were joined into a single term $\boldsymbol{r}_{ab}$, while the inner Jacobi distances $\boldsymbol{r}_{(I)}$ and $\boldsymbol{r}_{(II)}$ were also combined into $\boldsymbol{r}_{(I,II)}$; altogether leading to the correlation matrix shown in Fig. S6(a). The final correlation matrix employed in the main texts, which is shown in Fig. S6(b), derives from truncating the latter matrix into only the seven coordinates discussed there, i.e., R_0, z, $\boldsymbol{\gamma}$, $\boldsymbol{\Theta}$, $\boldsymbol{\Theta'}$ and $\boldsymbol{\Gamma}$. It should be noted that in the main text, we refer to R_0 as r because lowercase notation is used there to denote motions associated with the Zundel-core.

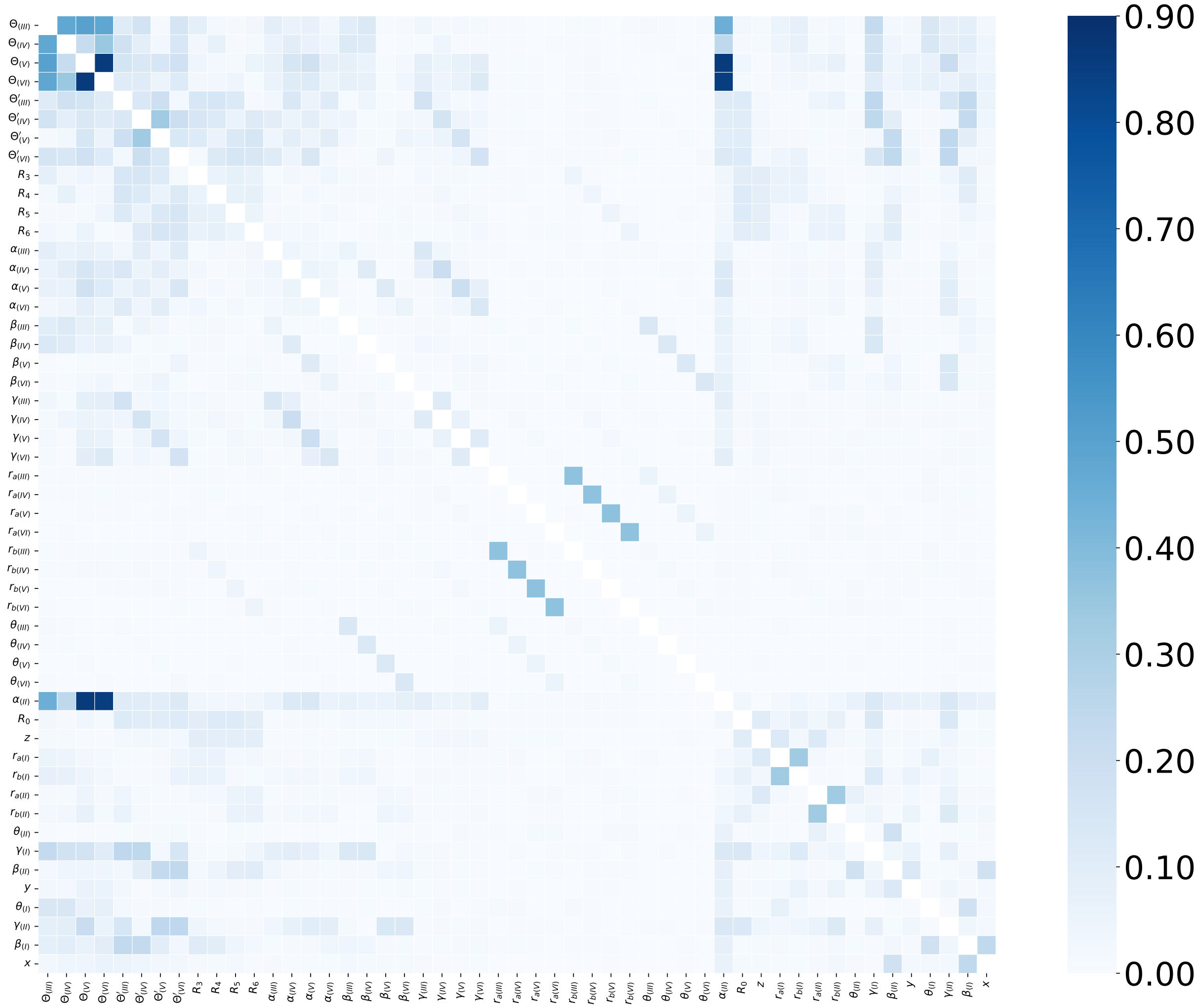

FIG. S4. Full-dimensional correlation matrix.

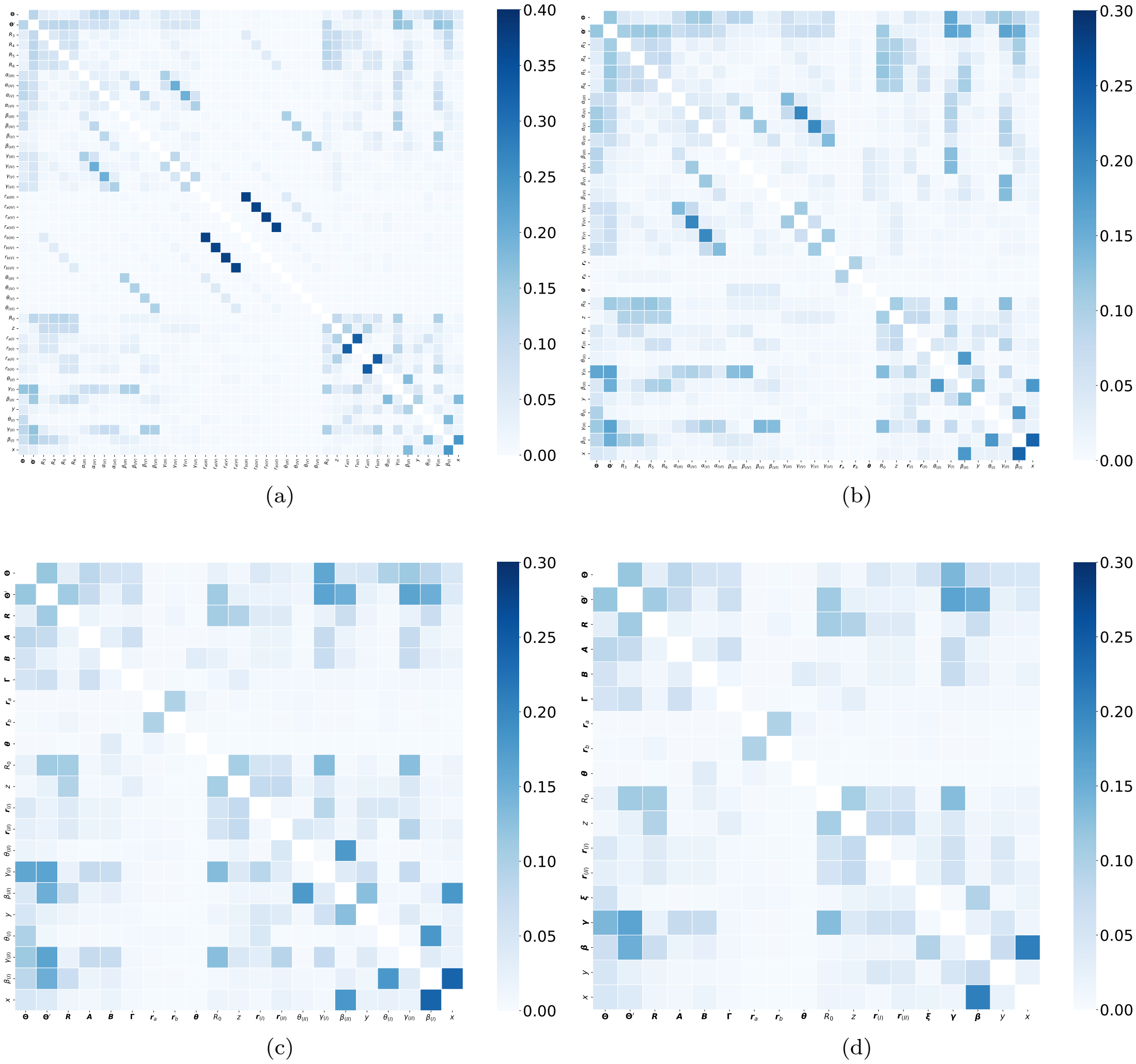

FIG. S5. Reduced dimensionality correlation matrices by averaging over groups of coordinates. (Note that coordinate labels may be too small to distinguish in print, but the resolution of the image is sufficient to identify them by zooming in.)

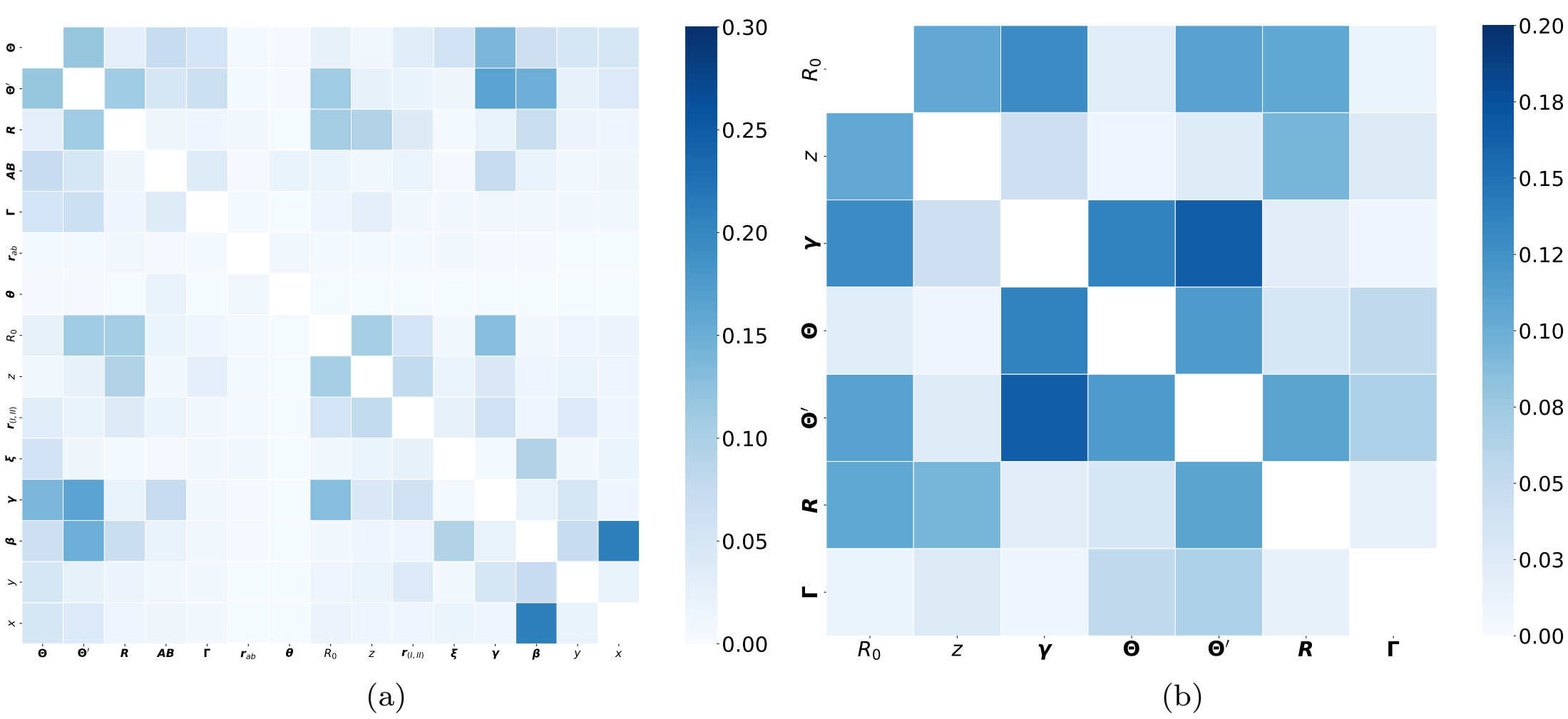

FIG. S6. Lowest dimensionality correlation matrices obtained by averaging over groups of coordinates; note that R_0 corresponds to r in the main text.

S4. WAVEFUNCTION REPRESENTATION AND PRIMITIVE BASIS SET

In the multi-configuration time-dependent Hartree (MCTDH) method, the nuclear wavefunction is expanded in a time-dependent basis of configurations, which are also in turn represented as linear combinations, but now in a time-independent basis. This can be seen as a two-layer approach, i.e., with the top layer coefficients accounting for the correlation between products of time-dependent basis functions, and the second (last) layer describing the expansion of the time-dependent basis. The time-dependent variational principle then yields equations of motion for these parameters. However, despite the inherent advantages derived from employing a time-dependent variational basis, the scaling with respect to the system size remains exponential in MCTDH. Therefore, here we employ the so-called multi-layer (ML) extension of MCTDH, ML-MCTDH, which allows for the construction of very compact representations of the many-body wavefunction, and consequently significant reductions in the computational cost.

The ML-representation of the wavefunction employed in the extended Zundel complex is shown in Fig. S7. Here, rectangles at the bottom denote the underlying physical coordinates — the internal coordinates described in Sec. S2 — and circles on upper layers describe logical coordinates. Labels on top of connective lines show the number of basis functions in the corresponding expansion.

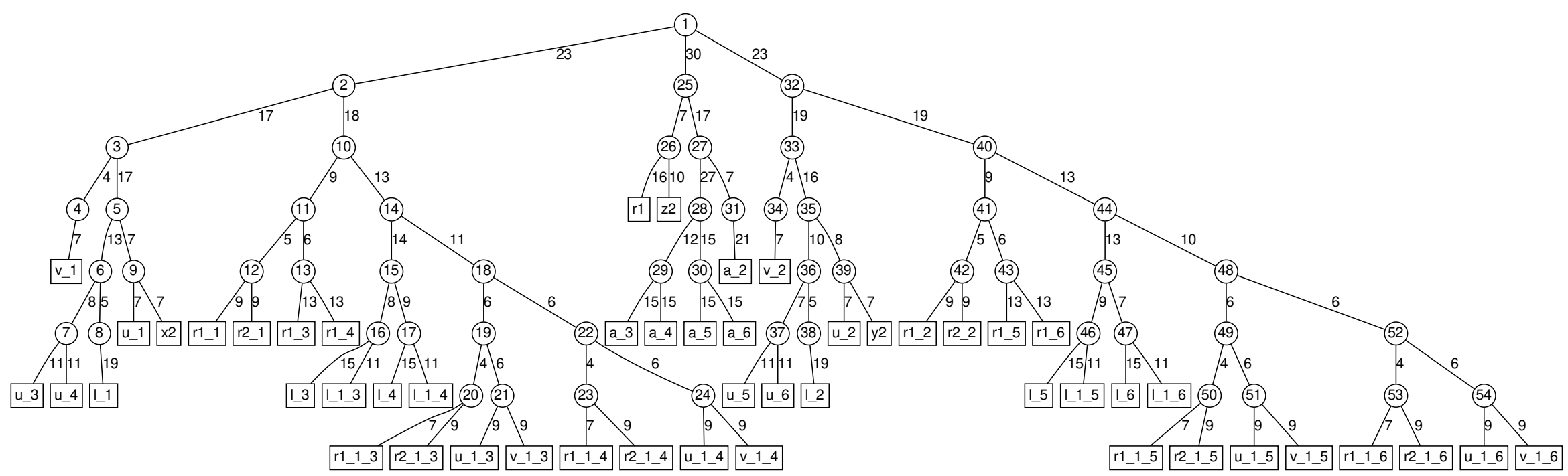

FIG. S7. Multi-layer tree used in the MCTDH calculations for eZ, $H_{13}O_6{}^{+}$. The total size of the wavefunction tensor is $8.2{\cdot}10^4$ complex numbers for the ground state imaginary-time relaxation.

The choice of one-dimensional basis functions for each physical coordinate is outlined in Table S1, detailing the coordinate ranges and number of grid points used for each of the 51 physical coordinates (i.e., time-independent or primitive basis).

S5. POTENTIAL ENERGY AND DIPOLE MOMENT SURFACE FITS

As implemented in the Heidelberg package[49], the MCTDH method requires a PES in a sum-of-products form. Therefore, a pre-processing fitting step is necessary when an analytical expression in this form is not available. In this work, the potential energy and dipole moment were fitted into a numerical representation using a canonical polyadic decomposition (CPD)[73].

The low-dimensional basis functions were defined on the coordinates corresponding to those of the bottom layer of the wavefunction tree (see Fig. S7) and the decomposition was created using a Monte Carlo variant of the alternating least squares algorithm. Further details of the algorithm can be found in Ref. [74]. The surface fitting needs to be performed in the internal dynamical coordinates, but the NN-PES and NN-DMS routines require Cartesian coordinates to calculate the respective property. Therefore, we interlinked the TANA program with the fitting program to enable transformation between the two sets of coordinates.

The potential energy and dipole moment components (x, y and z) required 1536 low-dimensional terms. Figure 8(a) and 8(b) show the corresponding root-mean-square error (RMSE) as a function of the number of iteration steps. These were fitted using a total of $1.6 \cdot 10^6$ sampling points that were divided into training ($5.4 \cdot 10^5$) and validation ($1.1 \cdot 10^6$) datasets. For accuracy and consistency, these quantum distributions were sampled from explicit PIQTB simulations using the same NN-PES at temperatures of 1, 5, 10, 20 and 50 K to include also significant fluctuations of the nuclear skeleton; recall that MCTDH is a ground-state quantum simulation method that solves the time-dependent Schrödinger equation formally at 0 K.

Coordinate	Basis	Size	x_0	x_f	Units
R_0	ho	16	4.0	6.6	au
R_1, R_2, R_3, R_4	sin	13	4.2	7.2	au
$r_{a(I)}$, $r_{a(II)}$	ho	9	2.2	3.8	au
$r_{b(I)}$, $r_{b(II)}$	ho	9	0.5	1.8	au
$\theta_{(I)}$, $\theta_{(II)}$	sin	7	-0.5	0.5	
$\alpha_{(II)}$	exp	21	0	2π	rad
$\beta_{(I)}$, $\beta_{(II)}$	sin	7	-0.5	0.5	
$\gamma_{(I)}$, $\gamma_{(II)}$	sin	19	-1.8	1.8	rad
x, y	ho	7	-0.9	0.9	au
z	ho	10	-1.2	1.2	au
$r_{a(III)}$, $r_{a(IV)}$, $r_{a(V)}$, $r_{a(VI)}$	ho	7	2.0	4.0	au
$r_{b(III)}$, $r_{b(IV)}$, $r_{b(V)}$, $r_{b(VI)}$	ho	9	0.5	1.8	au
$\theta_{(III)}$, $\theta_{(IV)}$, $\theta_{(V)}$, $\theta_{(VI)}$	sin	9	-0.5	0.5	
$\alpha_{(III)}$, $\alpha_{(IV)}$, $\alpha_{(V)}$, $\alpha_{(VI)}$	exp	15	0	2π	rad
$\beta_{(III)}$, $\beta_{(IV)}$, $\beta_{(V)}$, $\beta_{(VI)}$	sin	9	-0.8	0.8	
$\gamma_{(III)}$, $\gamma_{(IV)}$, $\gamma_{(V)}$, $\alpha_{(VI)}$	sin	11	$\pi - 1.8$	$\pi + 1.8$	rad
$\Theta_{(III)}$, $\Theta_{(IV)}$, $\Theta_{(V)}$, $\Theta_{(VI)}$	ho	11	1.3	3.1	rad
$\Theta'_{(III)}$, $\Theta'_{(IV)}$, $\Theta'_{(V)}$, $\Theta'_{(VI)}$	ho	15	1.3	4.9	rad

TABLE S1. Primitive coordinates, discrete variable representation (DVR) basis type and the corresponding grid parameters. ho, sin and exp indicate harmonic oscillator, sine, and exponential DVRs, respectively; note that R_0 corresponds to r in the main text.

S6. N-MODE REPRESENTATION OF DIPOLE SURFACES

Fully quantum-mechanical wavefunction computations on such a large molecular system (such as 51 dimensions for eZ) presents significant convergence challenges. This difficulty arises in part from the extensive correlation between vibrational states within the nuclear wavefunction. However, it is not necessary to propagate all of these simultaneously. Just as the PES can be decomposed into sums over terms that depend on subsets of coordinates, a similar decomposition can be applied to the dipole moment operator,

$$|\Psi_\mu\rangle = \hat{\mu}|\Psi_0\rangle \tag{8}$$

$$= \left[\sum_{d=1}^{N_d} \hat{\mu}_d(\{\vec{Q}\}_d)\right]|\Psi_0\rangle = \sum_{d=1}^{N_d} |\Psi_\mu^{(d)}\rangle \ . \tag{9}$$

This partitioning of the dipole surfaces into N_d terms, each depending on a specific subset of coordinates $\{\vec{Q}\}_d$, enables the definition of independent wavefunction propagations corresponding to excitations of particular groups of degrees of freedom. The vibrational spectrum is then obtained in the usual manner, via the Fourier transform of the dipole autocorrelation function,

$$a_\mu(t) = \langle\Psi_\mu(0)|\Psi_\mu(t)\rangle \ .$$

In this framework, both diagonal and off-diagonal (cross-correlation) contributions between the components $|\Psi_\mu^{(d)}\rangle$ contribute to the total autocorrelation function. Their evaluation within the ML-MCTDH method is straightforward. Naturally, the accuracy of the resulting spectrum depends sensitively on the chosen partitioning scheme, which required careful benchmarking for eZ.

Figure S9 shows the individual contributions of the four dipole partitions employed in this work ($N_d = 4$). The individual spectra are plotted to scale, reflecting their respective contributions to the total spectrum.

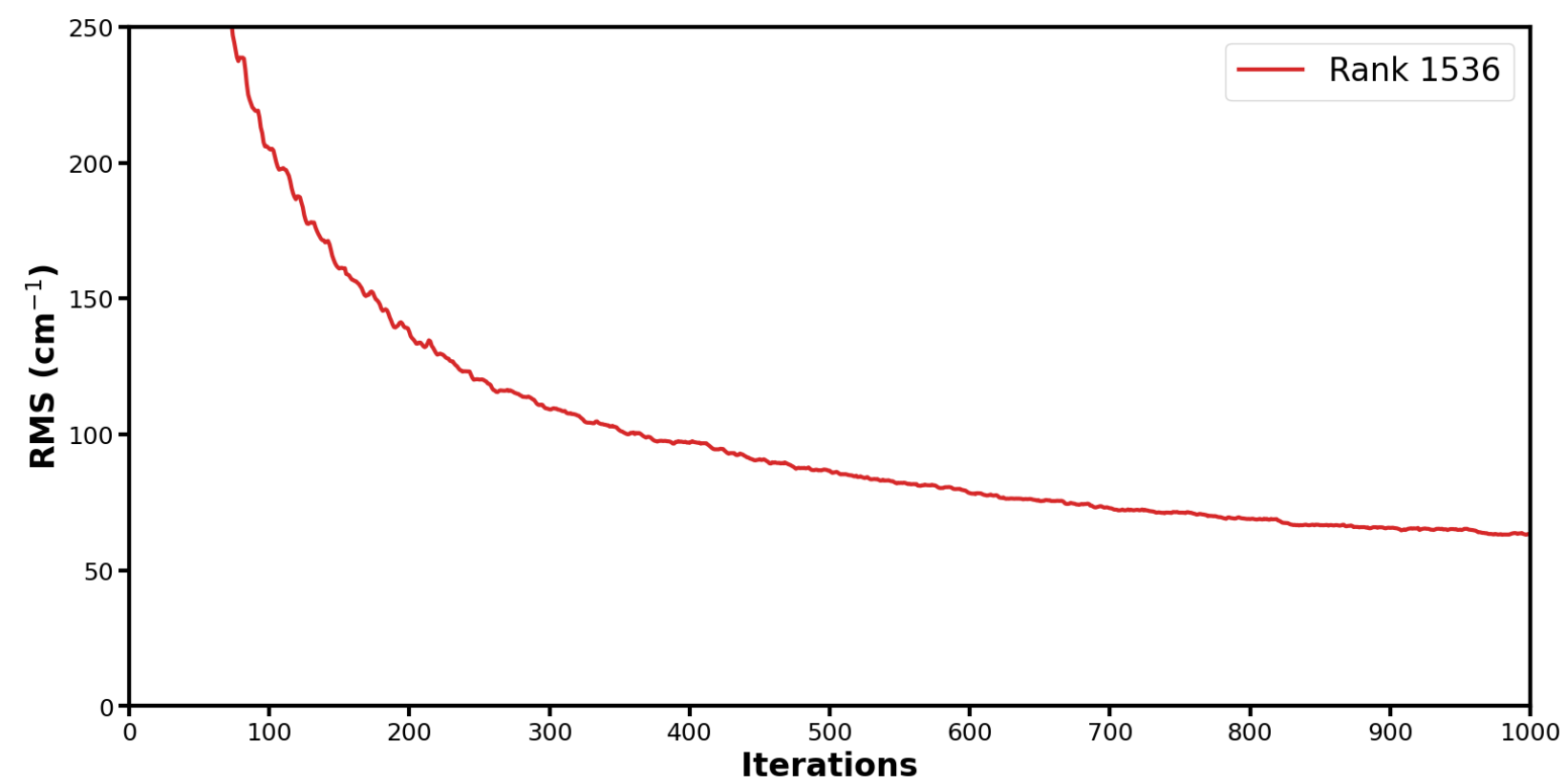

(a) Potential energy surface.

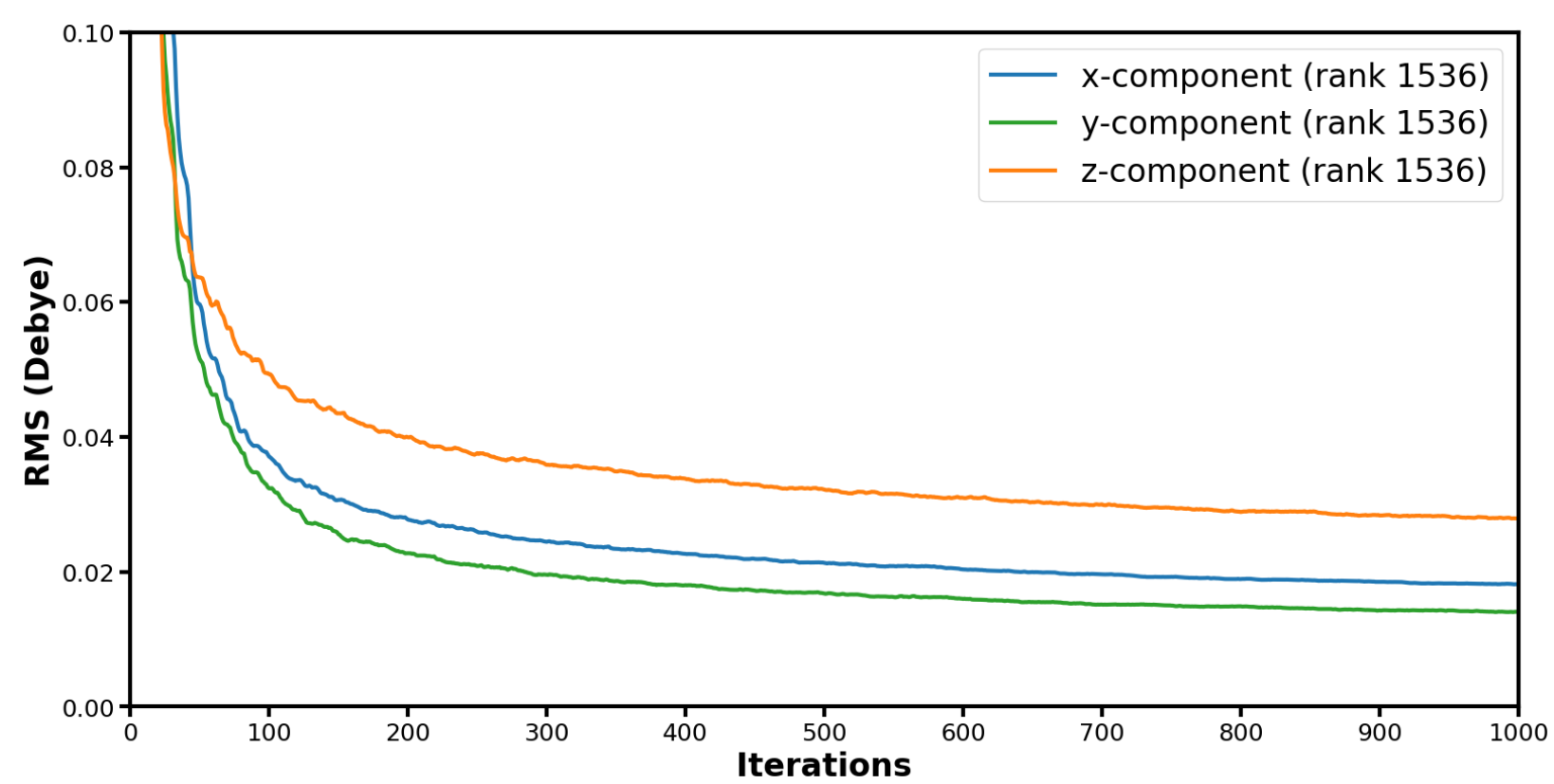

(b) Dipolar moment surface.

FIG. S8. Root-mean-square error (RMSE) as a function of the number of iteration steps for the sum-of-products fits.

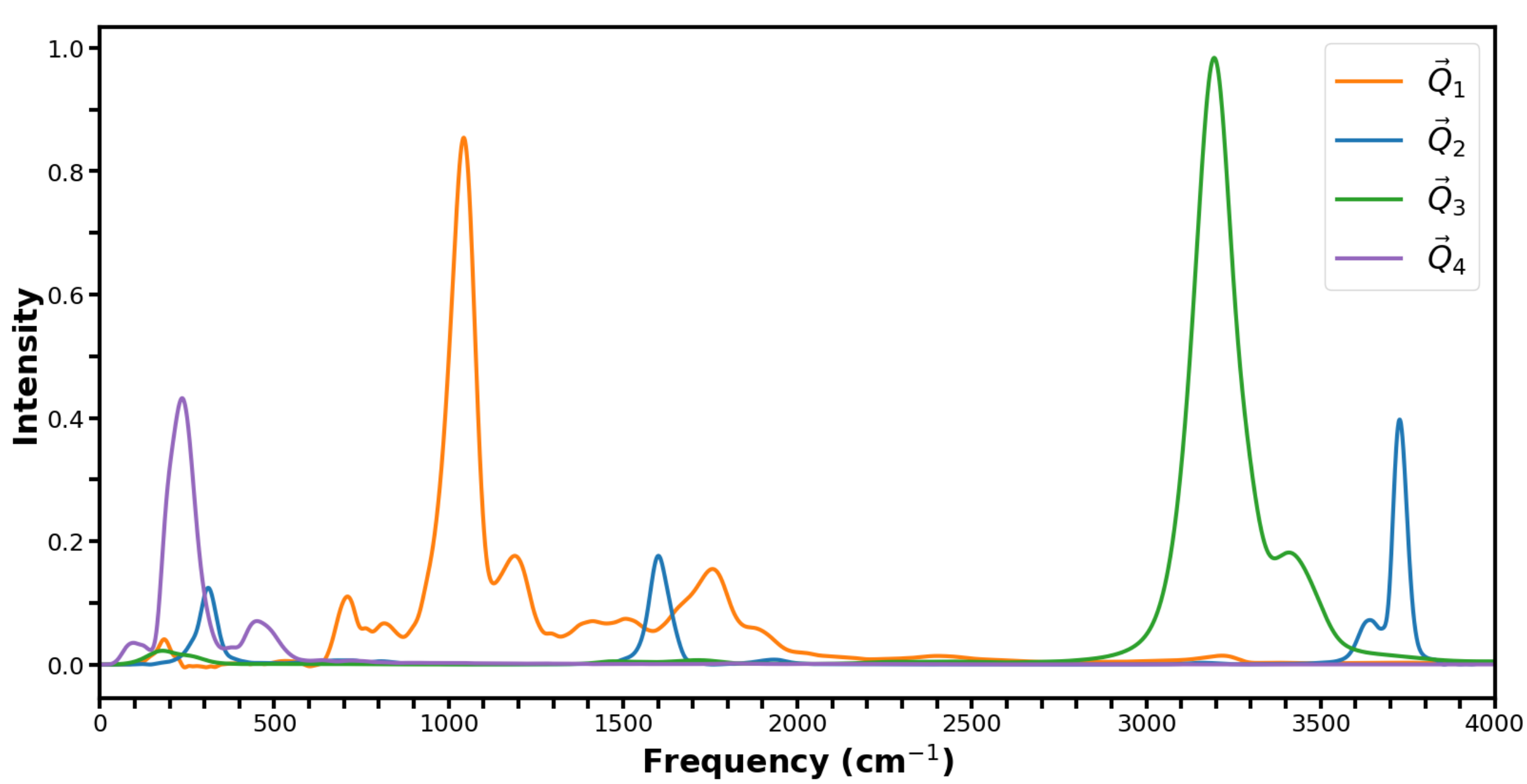

FIG. S9. Individual calculated spectra of eZ corresponding to the dipole moment partitions $\vec{Q}_1 = \{R_0, \alpha_{(II)}, z\}$ (orange), $\vec{Q}_2 = \{r_a, r_b, \theta, \beta\}_{\mathrm{III,IV,V,VI}}$ (blue), $\vec{Q}_3 = \{r_a, r_b, \theta\}_{\mathrm{I,II}}$, $\{R_1, R_2, R_3, R_4\}$ (green) and $\vec{Q}_4 = \{\gamma_{(III)}, \gamma_{(IV)}, \gamma_{(V)}, \gamma_{(VI)}\}$ (purple).

S7. ASSIGNMENT OF THE IR SPECTRUM OF THE EXTENDED ZUNDEL IN THE GAS PHASE

Figure 3a in the mansucript shows the calculated absorption spectrum of the eZ complex ($H_{13}O_6{}^+$) along with the experimental action spectrum reported in Ref. [22], specifically the spectrum labeled as (C) in Figure 3 (on page 8268) therein. The latter has recently been obtained via isomer-selective double-resonance population labeling spectroscopy of messenger-tagged $H^+(H_2O)_6 \cdot H_2$ complexes[22]. This exhibits approximately ten absorption bands from 280 to 3900 cm^{-1}, several of which are significantly broadened. The overall agreement between the calculated and experimental IR spectra is excellent; the gray line shades out an ill-resolved frequency window given the flat 2000-3000 cm^{-1} baseline reported in Figure 5e of Ref. [21] based on Ar tagging spectroscopy. In particular, we observe that all spectral features are accurately reproduced across the full spectral range without the need for any *ad hoc* adjustments.

The resolution of the theoretical spectrum is approximately 15 cm^{-1}, limited by the 2 ps duration of the dipole-dipole correlation function. Extending the correlation time would require longer propagations, which is computationally prohibitive on current hardware for a wavefunction of this size. Consequently, the peak positions are considered accurate to within a few tens of cm^{-1} and are rounded to the nearest 10 cm^{-1} when listed or discussed. Table S2 reports the corresponding calculated peak positions, together with the corresponding experimental values following the labeling convention established in Ref. [22].

In order to aid the interpretation of the observed bands, we selected key nuclear coordinates and performed localized vibrational excitations. These excitations were assigned by comparison with the spectral positions of carefully constructed test states generated by applying a specific coordinate operator $\hat{O}$ to the ground-state wavefunction $|\Psi_0\rangle$,

$$|\Phi_{Test}\rangle = N\hat{O}\,|\Psi_0\rangle \quad , \tag{10}$$

where N is a normalization constant. The coordinate operators $\hat{O}$ are the same as the definitions given in Sec. S2, which allowed for the following peak assignments.

	Theory			Experiment [22]	
Solvation layer	Assignment	$\hat{O}$	MCTDH	Label	IR^2MS^2
Outer	O-O str.	R	240	a'_{19}	279
Outer	rocking	β	340	a''_{18}	(374)
Outer	wagging	γ	450	a'_{17}	469
Outer	libration	Θ	710	a'_{16}	708
Inner	wagging	γ	810	a'_{15}	~805
Inner	proton str.	z	1040	a'_{14}	1050
Outer	bending	$r_a - r_b$	1600	a'_{11}	1618
Inner	bending	$r_a - r_b$	1760	a'_{10}	1759
Inner	sym. str.	$r_a + r_b$	3190	a'_6	3167
Outer	sym. str.	$r_a + r_b$	3640	a'_4	3651
Outer	asym. str.	θ	3730	a'_2	3737
			3820	a'_1	3827

TABLE S2. Assignments, operators and peak positions in cm^{-1} of the vibrational bands observed in the IR spectra of $H^+(H_2O)_6$. Theoretical results are compared with experimental values derived from IR^2MS^2 spectroscopy probing at 3159 cm^{-1} of messenger-tagged $H^+(H_2O)_6 \cdot H_2$ gas phase clusters.[22] Experimental values in parentheses were not explicitly reported in Ref. [22], but were instead estimated via digitization of the published spectrum.

The lowest-frequency band in the theoretical spectrum, which is only partially covered by experiment, corresponds to oxygen-oxygen stretching motion involving the second solvation shell (dashed orange). Adjacent to this peak, there are two low-intensity bands between 390 and 600 cm^{-1} that also correspond to outer water motions (dashed), i.e., rocking and wagging. The two overlapping peaks located between 600 and 900 cm^{-1}, however, involve outer water librations (dashed purple) and inner wagging motion (continuous blue), respectively. It is worth highlighting that the wagging signals in the bare Zundel are observed at much lower frequency, about 100 cm^{-1}.

The very prominent unimodal peak at 1040 cm^{-1} is assigned to the dynamics of the central proton that forms the strong centered H-bond $H_2O\cdots H^+\cdots OH_2$, see the eZ complex inset in Fig. S1. This peak differs significantly from the previous cases, as the proton transfer dynamics along the oxygen-oxygen axis (more precisely the z coordinate) is strongly coupled to several other motions, resulting in a very broad band (yellow) that reaches up to 2000 cm^{-1}. Nonetheless, most of these coupled motions could be identified. More precisely, the low-intensity signal observed at 1400 cm^{-1} (light and dark blue) is associated with the motion of the central proton perpendicular to the oxygen-oxygen bond (namely in the xy plane), whilst the wide band at 1760 cm^{-1} (red) relates to the bending vibration of the inner water molecules. Meanwhile, the contrasting sharp peak at 1600 cm^{-1} (red) is also attributed to bending vibrations; however, it now relates to the outer water molecules (dashed).

In the high-frequency region of the spectrum, the high-intensity band spanning about 3000 to 3600 cm^{-1} is associated with the stretching vibrations of the inner protons. These protons donate the four H-bonds of the Zundel core $H_2O\cdots H^+\cdots OH_2$ to the solvating water molecules. Finally, the two sharp peaks at very high frequencies of 3652 and 3730 cm^{-1} are assigned to the symmetric and asymmetric stretching modes of the outer OH bonds, respectively. These are not involved in H-bonding as they correspond to dangling or free OH bonds.

S8. POTENTIAL ENERGY CUTS AND REDUCED DIMENSIONALITY CALCULATIONS

Freezing specific coordinates was achieved by removing from the Hamiltonian all differential operators associated with such variables while maintaining Hermiticity, and fixing their position to the corresponding expectation value in the ground vibrational state of the full-dimensional system. A potential energy surface including sampling points at large R_4 distances (referred to as R_{const} in the main text) was refitted for this purpose. Also, the number of DVR points for R_4 was increased proportionally, by a factor of three, from 13 to 39. For each of the curves illustrated in Figure 5 of the main text, a new imaginary time-relaxation and posterior test-state propagations were performed for the corresponding lower-dimensional cut.

**Grid point**	$\boldsymbol{R_4}$ ($\boldsymbol{R_{\text{const}}}$)	$\boldsymbol{z}$	$\boldsymbol{R_0}$	$\boldsymbol{R_1}$	$\boldsymbol{R_2}$	$\boldsymbol{R_3}$
Unconstrained	2.711	0.000	2.426	2.710	2.709	2.711
4	2.639	0.015	2.423	2.720	2.716	2.709
5	2.780	-0.070	2.428	2.684	2.684	2.783
6	2.921	-0.144	2.437	2.652	2.658	2.795
7	3.062	-0.203	2.449	2.625	2.637	2.793
8	3.202	-0.244	2.460	2.603	2.619	2.808
11	3.617	-0.250	2.458	2.591	2.605	2.789
13	3.894	-0.289	2.474	2.546	2.572	2.788
14	4.032	-0.302	2.479	2.541	2.569	2.783
15	4.169	-0.308	2.480	2.541	2.564	2.797
17	4.453	-0.332	2.487	2.536	2.558	2.801
20	4.874	-0.360	2.498	2.534	2.559	2.797
26	5.703	-0.391	2.512	2.530	2.547	2.778
39	7.515	-0.416	2.523	2.523	2.541	2.783

TABLE S3. Ground state equilibrium distances (Å) for a few selected coordinates (z, R_0, R_1, R_2 and R_3) along a scan along coordinate R_4 (see Fig. S2a for their definition; note that R_0 corresponds to r in the main text).

Table S3 summarizes the expectation value of the ground vibrational state for a few selected coordinates. These show how, as R_4 increases progressively from 2.6 to 7.5 Å, the proton transfer coordinate (z) is increasingly localized toward one of the oxygen atoms. Meanwhile, the distance R_0 (corresponding to r in the main text) shows a modest increase from 2.4 Å at equilibrium to approximately 2.5 Å at the largest value of R_4. Importantly, both coordinates R_3 and R_4 also show a trend, but in this case more pronounced and toward decreasing values, which also stabilizes near 2.5 Å. Therefore, indicating the formation of an Eigen-like structure with a central hydronium core that is symmetrically H-bonded to three surrounding water molecules, i.e., $H_3O^+(H_2O)_3$. On the other hand, the coordinate R_3 remains instead relatively constant over much of the scan with fluctuating values in between 2.7 and 2.8 Å.

S9. CROSS-CORRELATION ANALYSIS BETWEEN LOCALIZED EXCITATIONS: UNRAVELING THE PROTON TRANSFER AND INNER-WATER BENDING COUPLING

The coupling between zeroth-order excitations represented by $|\psi_1\rangle$ and $|\psi_2\rangle$ can be established by analyzing their cross-correlation function,

$$
\begin{aligned}
C_{12}(t) &= \langle \psi_1(0) \,|\, e^{-i\hat{H}t/\hbar} \,|\, \psi_2(0)\rangle && (11)\\
&= \sum_{j,l} c_j^{(1)*}(0)\; c_l^{(2)}(t) e^{-i\omega_l t}\;\; \langle \phi_j|\phi_l\rangle && (12)\\
&= \sum_{j} c_j^{(1)*}(0)\; c_j^{(2)}(0) e^{-iw_j t}. && (13)
\end{aligned}
$$

Here, $|\phi_j\rangle$ denotes the energy eigenstates of the system. One sees how the cross-correlation function corresponds to the sum of products of coefficients of the eigenstates spanning the wavepacket of both test states, times a corresponding time-dependent phase. Since the initial zeroth-order states can be taken to be real, we remove the star from the bra-side coefficients at $t = 0$, $c_j^{(\nu)*}(0) = c_j^{(\nu)}(0)$. The (inverse) Fourier transform of $C_{12}(t)$ can be performed, yielding

$$
\begin{aligned}
C_{12}(\omega) &= \int_{-\infty}^{\infty} \left[\sum_j c_j^{(1)}(0)\; c_j^{(2)}(0) e^{-iw_j t} \right] e^{iwt} dt && (14)\\
&= 2\pi \sum_j c_j^{(1)}\; c_j^{(2)} \delta(\omega - \omega_j). && (15)
\end{aligned}
$$

Hence, the cross-correlation between zeroth-order states in the frequency domain at frequency ω_j corresponds to the product of the expansion coefficients of eigenstate $|\phi_j\rangle$ with energy $E_j = \hbar\omega_j$. Only if the eigenstate participates in both zeroth-order excitations does it appear in the spectrum, otherwise $C_{12}(\omega_j) = 0$. Clearly, $C_{12}(\omega)$ is a real function if both zeroth-order states are real, and it can have positive and negative values depending on the relative sign of the expansion coefficients of $|\phi_j\rangle$ at each $\omega = \omega_j$. The absolute value of $C_{12}(\omega)$, however, is not meaningful because multiplying any of the test states with -1 results in $C_{12}(\omega) \to -C_{12}(\omega)$, while leaving the physical properties of such a state unaltered.

We proceed now to analyze the cross-correlation between the $|\psi_z\rangle$ and $|\psi_u\rangle$ excitations ($|\psi_{\mathsf{pt}}\rangle$ and $|\psi_{\mathsf{u\text{-}bend}}\rangle$ in the main text). These are the central proton and *ungerade* bending of the inner water molecules, respectively. This coupling underlies the joint response of these coordinates, as depicted in Figure 5b of the main text. Both zeroth-order states were generated by applying a suitable operator to the absolute ground state of the system, $|\psi_\nu\rangle = \mathcal{O}_\nu\,|\Psi_0\rangle$.

Figure S10 shows the Fourier transform of the cross-correlation function between the $|\psi_z\rangle$ and each of the $|\psi_u\rangle$ and $|\psi_{u,out}\rangle$ for the symmetric eZ system, where $|\psi_{u,out}\rangle$ is the bending motion of the outer water molecules. As expected, the inner bending shows a significant correlation with $|\psi_z\rangle$ as compared to outer bendings, which present nearly null contribution. The very prominent unimodal peak assigned to the central proton mode appears at 1040 cm^{-1}, while the broad band centered at 1750 cm^{-1} can be mostly connected with the asymmetric bending of the inner water molecules. It is important to note here, that the eigenstates at these frequencies contain contributions from both zeroth-order states, or equivalently, the zeroth-order states are spanned by the eigenstates at both frequencies. Whereas the absolute sign of the cross-correlation function in frequency domain is irrelevant as explained above, the relative sign is compatible with the following linear combinations of eigenstates for the zeroth-order states,

$$
\begin{aligned}
\psi_z &\approx c_{\mathsf{low}}^{(z)} \phi_{\mathsf{low}} + c_{\mathsf{high}}^{(u)} \phi_{\mathsf{high}} && (16)\\
\psi_u &\approx c_{\mathsf{low}}^{(z)} \phi_{\mathsf{low}} - c_{\mathsf{high}}^{(u)} \phi_{\mathsf{high}} && (17)\\
& && (18)
\end{aligned}
$$

or

$$\psi_z \approx -c^{(z)}_{\mathsf{low}}\phi_{\mathsf{low}} + c^{(u)}_{\mathsf{high}}\phi_{\mathsf{high}} \tag{19}$$

$$\psi_u \approx c^{(z)}_{\mathsf{low}}\phi_{\mathsf{low}} + c^{(u)}_{\mathsf{high}}\phi_{\mathsf{high}} \tag{20}$$

$$\tag{21}$$

where $\phi_{\mathsf{low/high}}$ refer to the the eigenstates with the lower and higher energy in the spectrum, respectively. Note also that this is a low resolution picture of the coupling, and that a long time-correlation might unravel finer structures in the frequency domain involving more eigenstates. Therefore, the different sign of both main peaks derives from the fact that the spectrally active eigenstates are linear combinations of both zeroth-order states with opposing relative signs, i.e. the positive and negative linear combination of the zeroth-order motions.

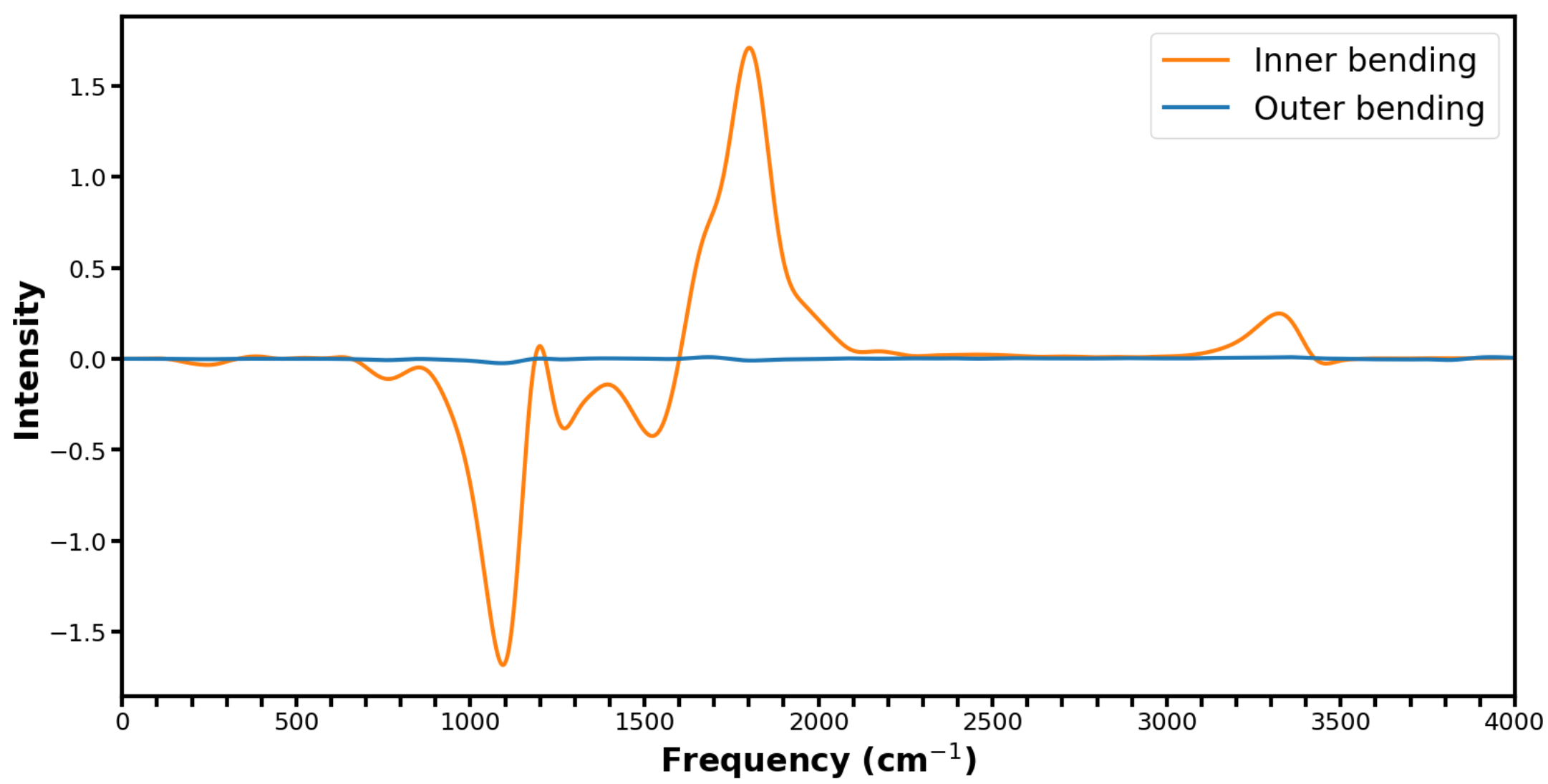

FIG. S10. Fourier transforms of the cross-correlation function between the time-dependent proton transfer (z) test-state and the inner (orange) and outer (blue) bending motions.

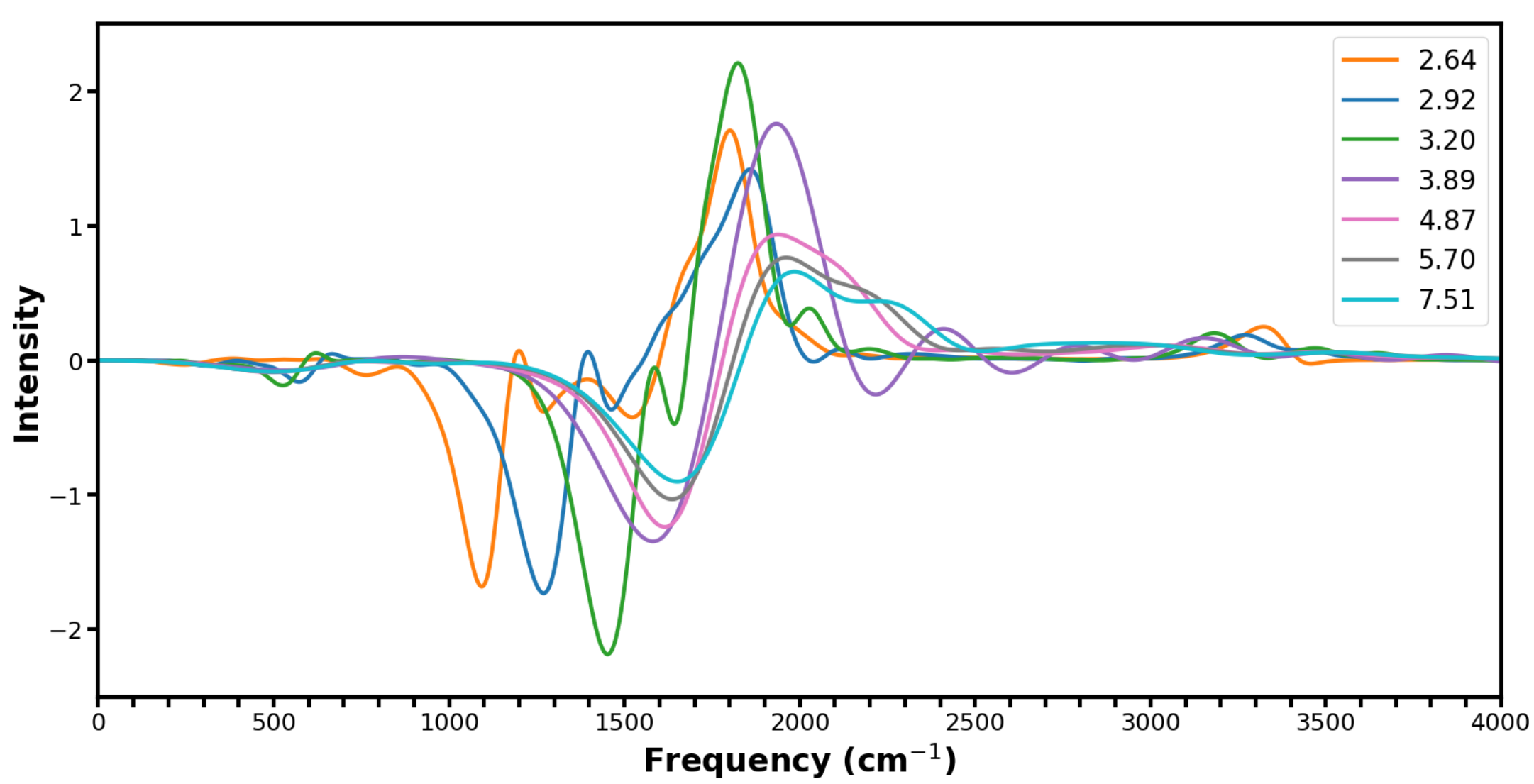

FIG. S11. Fourier transforms of the cross-correlation function between the time-dependent proton transfer (z) and the inner bending motions at different values of R_4 (corresponding to R_{const} in the main text.)

Next, Fig. S11 illustrates the cross-correlation spectra between $|\psi_z\rangle$ and the $|\psi_u\rangle$ along scanned values of the outer water distance R_{const}, the same time-propagations corresponding to the traces in Figure 5b in the main text. A prominent feature of this analysis is that the low-frequency peak shifts to the blue very rapidly as the asymmetry is increased from $R_{\mathsf{const}} = 2.64$ to 3.20 Å, as the orange, dark blue and green peaks indicate. The corresponding peaks at higher energy are almost not moving, in contrast. This indicates that, as the asymmetry increases, the proton transfer mode quickly shifts to the blue, whereas the asymmetric inner-water bending is quite insensitive to this increasing asymmetry. Eventually, the position of the $|\psi_z\rangle$ zeroth-order crosses $|\psi_u\rangle$. In Figure 5b in the main text, this results in a doublet structure in the response (autocorrelation) of $|\psi_z\rangle$. The cross-correlation with $|\psi_u\rangle$ results in the bi-modal structure for the largest distances with peak-to-peak splitting of about 400 cm^{-1} for the largest R_{const} distances.

S10. COUNTERION IMPACT IN THE LIQUID STATE

The second solvation shell of the excess proton in bulk water, H^+(aq), is known to occur at roughly 3 Å according to the respective O–H^+ RDF (see right panels of Figures 16 and 17 in Ref. [75]). The cylindrical distance distribution function (obtained from ab initio molecular dynamics (AIMD) [76] as detailed below) between the excess proton H^+ and the water oxygen atoms in 2 M HCl solution in the direction of the counterion Cl^- (red line) features a prominent peak starting at the same distance of ≈ 3 Å, which is very slowly decaying toward distances of 4 Å and beyond, corresponding to enhanced probability to find second solvation shell water molecules in between the excess proton and the counterion at these distances. The distance and radial distribution functions between H^+ and Cl^- in 2 M HCl solution (green and blue lines) show a pronounced peak that extends from roughly 3.2 to 4.0 Å. Taken together, this implies that Cl^- enters the second solvation shell of the aqueous proton H^+(aq) with a high probability in 2 M aqueous HCl solution. This counterion effect certainly impacts on the properties of the local solvation environment of the excess proton in such acidic solutions (with reference to the desired infinite dilution limit that hosts the "pristine aqueous proton"), thus also affecting its H-bond motifs, whereas this perturbation is rigorously absent in gas phase mimics of the hydrated proton as used herein.

Simulation Details: The system consists of one hydronium ion (H_3O^+), one chloride ion (Cl^-), and 27 water molecules (H_2O) in a periodic cubic supercell of 9.4 Å, corresponding to a 2 M aqueous HCl solution as used in 2D-IR spectroscopy experiments [12]. A 80 ps AIMD simulation [76] was performed in the NVT ensemble at 300 K following pre-equilibration using force field MD. The shown analysis was performed using the final 70 ps. The AIMD simulation employed the RPBE exchange–correlation functional with D3 dispersion correction (RPBE-D3) [77–79] in combination with Goedecker–Teter–Hutter (GTH) pseudopotentials [80–82] and a triple-zeta valence with double polarization (TZV2P) basis set as implemented in the CP2K simulation package [67, 68].

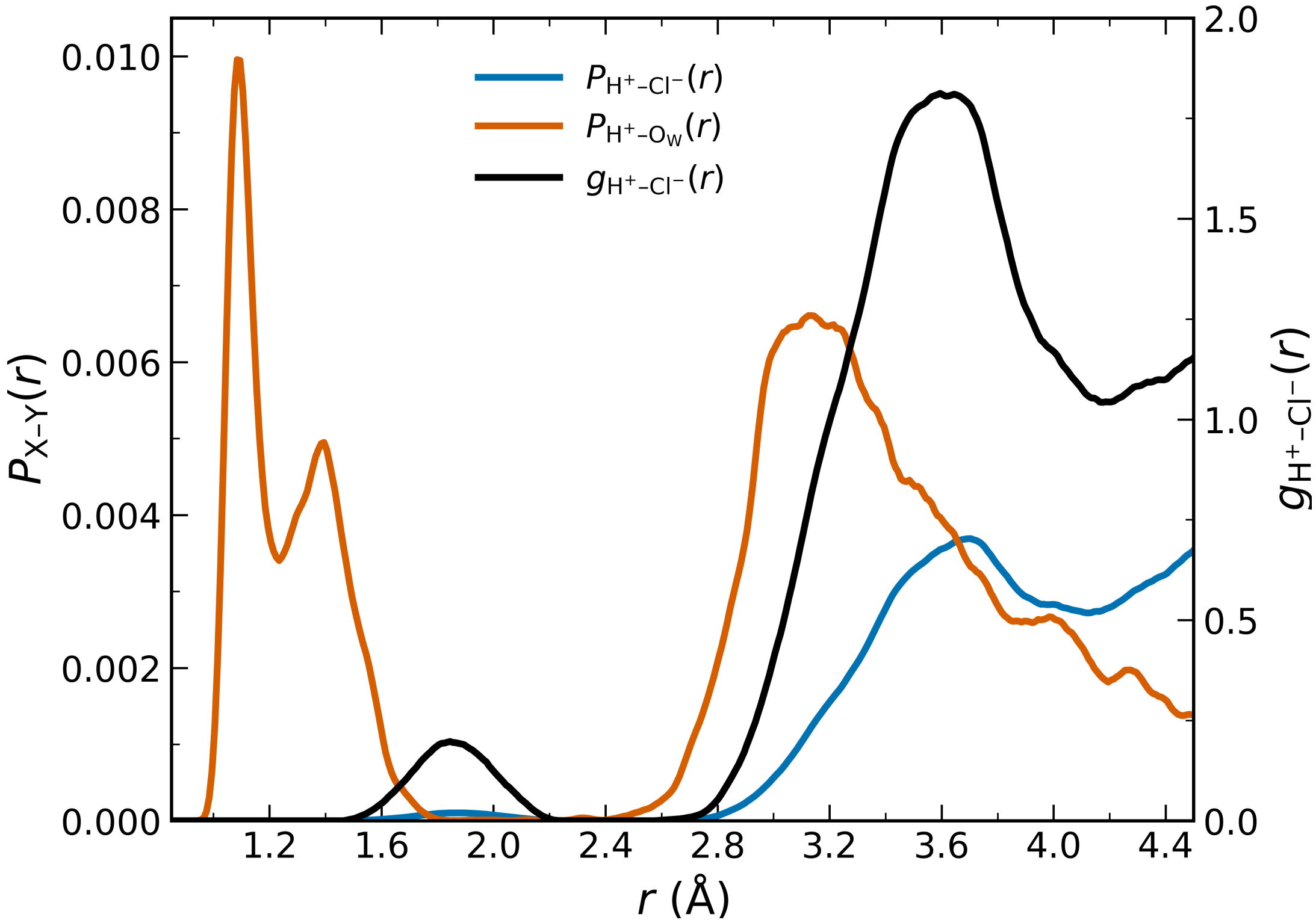

FIG. S12. Ion pairing in 2 M aqueous HCl solution as used in 2D-IR spectroscopy [12] to measure the IR response of the aqueous proton. Distance distribution function (left scale) of the chloride ion (Cl^-) relative to the excess proton (H^+) is shown in green while the corresponding radial distribution function (RDF, right scale) in blue. The distance distribution function of the oxygen atom of water molecules relative to the excess proton (red) was computed by selecting the oxygen atoms located within a cylindrical volume of 3 Å radius centered along the proton–chloride axis. The data have been obtained from AIMD simulations, see text.

[1] Zundel, G. & Metzger, H. Energiebänder der tunnelnden Überschuß–Protonen in flüssigen Säuren. Eine IR–spektroskopische Untersuchung der Natur der Gruppierungen $H_5O_2^+$. *Z. physik. Chemie (N.F. Frankfurt)* **58**, 225 (1968).

[2] Wicke, E., Eigen, M. & Ackermann, T. Über den zustand des protons (hydroniumions) in wäßriger lösung*. *Z. Phys. Chem.* **1**, 340–364 (1954).

[3] Xu, J., Zhang, Y. & Voth, G. A. Infrared spectrum of the hydrated proton in water. *J. Phys. Chem. Lett.* **2**, 81–86 (2010). URL http://dx.doi.org/10.1021/jz101536b.

[4] Kulig, W. & Agmon, N. A 'clusters-in-liquid' method for calculating infrared spectra identifies the proton-transfer mode in acidic aqueous solutions. *Nat. Chem.* **5**, 29–35 (2012).

[5] Biswas, R., Carpenter, W., Fournier, J. A., Voth, G. A. & Tokmakoff, A. IR spectral assignments for the hydrated excess proton in liquid water. *J. Chem. Phys.* **146**, 154507 (2017).

[6] Daly, Jr., C. A. *et al.* Decomposition of the Experimental Raman and Infrared Spectra of Acidic Water into Proton, Special Pair, and Counterion Contributions. *J. Phys. Chem. Lett.* **8**, 5246–5252 (2017).

[7] Napoli, J. A., Marsalek, O. & Markland, T. E. Decoding the spectroscopic features and time scales of aqueous proton defects. *J. Chem. Phys.* **148**, 222833 (2018). URL http://aip.scitation.org/doi/10.1063/1.5023704.

[8] Yu, Q., Carpenter, W. B., Lewis, N. H. C., Tokmakoff, A. & Bowman, J. M. High-Level VSCF/VCI Calculations Decode the Vibrational Spectrum of the Aqueous Proton. *J. Phys. Chem. B* **123**, 7214–7224 (2019).

[9] Carpenter, W. B. *et al.* Decoding the 2D IR spectrum of the aqueous proton with high-level VSCF/VCI calculations. *J. Chem. Phys.* **153**, 124506 (2020).

[10] Calio, P. B., Li, C. & Voth, G. A. Resolving the Structural Debate for the Hydrated Excess Proton in Water. *J. Am. Chem. Soc.* **143**, 18672–18683 (2021).

[11] Thämer, M., De Marco, L., Ramasesha, K., Mandal, A. & Tokmakoff, A. Ultrafast 2D IR spectroscopy of the excess proton in liquid water. *Science* **350**, 78–82 (2015). URL http://www.ncbi.nlm.nih.gov/pubmed/26430117.

[12] Fournier, J. A., Carpenter, W. B., Lewis, N. H. C. & Tokmakoff, A. Broadband 2D IR spectroscopy reveals dominant asymmetric $H_5O_2^+$ proton hydration structures in acid solutions. *Nat. Chem.* **10**, 932–937 (2018).

[13] Zeng, H. J. & Johnson, M. A. Demystifying the diffuse vibrational spectrum of aqueous protons through cold cluster spectroscopy. *Annu. Rev. Phys. Chem.* **72**, 667–691 (2021). URL http://dx.doi.org/10.1146/annurev-physchem-061020-053456.

[14] Schwarz, H. A. Gas phase infrared spectra of oxonium hydrate ions from 2 to 5 μ. *J. Chem. Phys.* **67**, 5525–5534 (1977).

[15] Okumura, M., Yeh, L. I., Myers, J. D. & Lee, Y. T. Infrared spectra of the solvated hydronium ion: Vibrational predissociation spectroscopy of mass-selected $H_3O^+{\cdot}(H_2O)_n{\cdot}(H_2)_m$. *J. Phys. Chem.* **94**, 3416–3427 (1990).

[16] Jiang, J.-C. *et al.* Infrared Spectra of $H^+(H_2O)_{5-8}$ Clusters: Evidence for Symmetric Proton Hydration. *J. Am. Chem. Soc.* **122**, 1398–1410 (2000).

[17] Asmis, K. R. *et al.* Gas-Phase Infrared Spectrum of the Protonated Water Dimer. *Science* **299**, 1375–1377 (2003).

[18] Headrick, J. M. *et al.* Spectral Signatures of Hydrated Proton Vibrations in Water Clusters. *Science* **308**, 1765–1769 (2005). Corrections and Clarifications: Science **309**, 1326 (2005).

[19] Hammer, N. I. *et al.* The vibrational predissociation spectra of the h5o2+rgn(rg=ar,ne) clusters: Correlation of the solvent perturbations in the free oh and shared proton transitions of the zundel ion. *J. Chem. Phys.* **122**, 244301 (2005). URL http://dx.doi.org/10.1063/1.1927522.

[20] Roscioli, J. R., McCunn, L. R. & Johnson, M. A. Quantum Structure of the Intermolecular Proton Bond. *Science* **316**, 249–254 (2007).

[21] Olesen, S. G., Guasco, T. L., Roscioli, J. R. & Johnson, M. A. Frontiers Article: Tuning the intermolecular proton bond in the $H_5O_2^+$ 'Zundel ion' scaffold. *Chem. Phys. Lett.* **509**, 89–95 (2011).

[22] Heine, N. *et al.* Isomer-selective detection of hydrogen-bond vibrations in the protonated water hexamer. *J. Am. Chem. Soc.* **135**, 8266–8273 (2013).

[23] Wolke, C. T. *et al.* Spectroscopic snapshots of the proton-transfer mechanism in water. *Science* **354**, 1131–1135 (2016). URL http://dx.doi.org/10.1126/science.aaf8425.

[24] Fagiani, M. R. *et al.* Gas phase vibrational spectroscopy of the protonated water pentamer: the role of isomers and nuclear quantum effects. *Phys. Chem. Chem. Phys.* **18**, 26743–26754 (2016).

[25] Vendrell, O., Gatti, F. & Meyer, H. Dynamics and Infrared Spectroscopy of the Protonated Water Dimer. *Angew. Chem. Int. Ed.* **46**, 6918–6921 (2007).

[26] Vendrell, O., Gatti, F. & Meyer, H. Strong Isotope Effects in the Infrared Spectrum of the Zundel Cation. *Angew. Chem. Int. Ed.* **48**, 352–355 (2009).

[27] Fournier, J. A. *et al.* Feature Article: Snapshots of Proton Accommodation at a Microscopic Water Surface: Understanding the Vibrational Spectral Signatures of the Charge Defect in Cryogenically Cooled $H^+(H_2O)_{n=2-28}$ Clusters. *J. Phys. Chem. A* **119**, 9425–9440 (2015).

[28] Yu, Q. & Bowman, J. M. High-Level Quantum Calculations of the IR Spectra of the Eigen, Zundel, and Ring Isomers of $H^+(H_2O)_4$ Find a Single Match to Experiment. *J. Am. Chem. Soc.* **139**, 10984–10987 (2017).

[29] Esser, T. K. *et al.* Deconstructing prominent bands in the terahertz spectra of h7o3+ and h9o4+: Intermolecular modes in eigen clusters. *J. Phys. Chem. Lett.* **9**, 798–803 (2018). URL http://dx.doi.org/10.1021/acs.jpclett.7b03395.

[30] Liu, J. *et al.* Towards complete assignment of the infrared spectrum of the protonated water cluster $H^+(H_2O)_{21}$. *Nat. Commun.* **12**, 6141 (2021).

[31] Schröder, M., Gatti, F., Lauvergnat, D., Meyer, H. & Vendrell, O. The coupling of the hydrated proton to its first solvation shell. *Nat. Commun.* **13**, 6170 (2022).
[32] Larsson, H. R. *et al.* State-resolved infrared spectrum of the protonated water dimer: revisiting the characteristic proton transfer doublet peak. *Chem. Sci.* **13**, 11119–11125 (2022).
[33] Gomez, A., Thompson, W. H. & Laage, D. Neural-network-based molecular dynamics simulations reveal that proton transport in water is doubly gated by sequential hydrogen-bond exchange. *Nat. Chem.* **16**, 1838–1844 (2024).
[34] Marx, D., Tuckerman, M. E., Hutter, J. & Parrinello, M. The nature of the hydrated excess proton in water. *Nature* **397**, 601–604 (1999).
[35] Tuckerman, M. E., Laasonen, K., Sprik, M. & Parrinello, M. . *J. Phys. Chem.* **99**, 5749 (1995).
[36] Markovitch, O. *et al.* Special pair dance and partner selection: Elementary steps in proton transport in liquid water. *J. Phys. Chem. B* **112**, 9456–9466 (2008). URL `http://dx.doi.org/10.1021/jp804018y`.
[37] Gatti, F. & Iung, C. Exact and constrained kinetic energy operators for polyatomic molecules: The polyspherical approach. *Phys. Rep.* **484**, 1–69 (2009).
[38] Gatti, F., Justum, Y., Menou, M., Nauts, A. & Chapuisat, X. Quantum-mechanical description of rigidly or adiabatically constrained molecular systems. *J. Mol. Spec.* **181**, 403 (1997).
[39] Chapuisat, X. & Iung, C. Vector parametrization of the N-body problem in quantum mechanics: Polyspherical coordinates. *Phys. Rev. A* **45**, 6217–6235 (1992).
[40] Gatti, F. *et al.* Vector parametrization of the n-atom problem in quantum mechanics. I. Jacobi vectors. *J. Chem. Phys.* **108**, 8804 (1998).
[41] Gatti, F., Iung, C., Menou, M. & Chapuisat, X. Vector parametrization of the n-atom problem in quantum mechanics. II. Coupled-angular-momentun spectral representations for four atom systems. *J. Chem. Phys.* **108**, 8821 (1998).
[42] Gatti, F. Vector parametrization of the n-atom problem in quantum mechanics. III. Separation into two sub-systems. *J. Chem. Phys.* **111**, 7225 (1999).
[43] Iung, C., Gatti, F., Viel, A. & Chapuisat, X. Vector parametrization of the n-atom problem in quantum mechanics. non-orthogonal coordinates. *Phys. Chem. Chem. Phys.* **1**, 3377 (1999).
[44] Mladenović, M. Rovibrational hamiltonians for general polyatomic molecules in spherical polar parametrization. I. Orthogonal representations. *J. Chem. Phys.* **112**, 1070–1081 (2000).
[45] Gatti, F., Munoz, C. & Iung, C. A general expression of the exact kinetic energy operator in polyspherical coordinates. *J. Chem. Phys.* **114**, 8275 (2001).
[46] Gatti, F. & Nauts, A. Vector parametrization, partial angular momenta and unusual commutation relations in molecular physics. *Chem. Phys.* **295**, 167–174 (2003).
[47] Gatti, F. & Iung, C. Exact and constrained kinetic energy operators in polyspherical coordinates. *J. Theor. Comp. Chem.* **2**, 507–522 (2003).
[48] Iung, C. & Gatti, F. Polyspherical parametrization of a n-atom system: Principles and applications. *Int. J. Quant. Chem.* **106**, 130 (2006).
[49] Worth, G. A., Beck, M. H., Jäckle, A., Vendrell, O. & Meyer, H.-D. The MCTDH Package, Version 8.2, (2000). H.-D. Meyer, Version 8.3 (2002), Version 8.4 (2007). O. Vendrell and H.-D. Meyer Version 8.5 (2013). H.-D. Meyer, Version 8.6 (2021). Versions 8.5 and 8.6 contain the ML-MCTDH algorithm. Current versions: 8.4.24, 8.5.17, and 8.6.5 (June 2023). See http://mctdh.uni-hd.de/ for a description of the Heidelberg MCTDH package.
[50] Vendrell, O., Gatti, F. & Meyer, H.-D. Strong isotope effects in the infrared spectrum of the Zundel cation. *Angew. Chem. Int. Ed.* **48**, 352 – 355 (2009).
[51] Vendrell, O. & Meyer, H.-D. A proton between two waters: insight from full-dimensional quantum-dynamics simulations of the $[H_2O\text{-}H\text{-}OH_2]^+$ cluster. *Phys. Chem. Chem. Phys.* **10**, 4692–4703 (2008).
[52] Vendrell, O., Gatti, F. & Meyer, H.-D. Dynamics and infrared spectroscopy of the protonated water dimer. *Angew. Chem. Int. Ed.* **46**, 6918–6921 (2007).
[53] Vendrell, O., Gatti, F., Lauvergnat, D. & Meyer, H.-D. Full dimensional (15D) quantum-dynamical simulation of the protonated water dimer I: Hamiltonian setup and analysis of the ground vibrational state. *J. Chem. Phys.* **127**, 184302 (2007).
[54] Vendrell, O., Gatti, F. & Meyer, H.-D. Full dimensional (15D) quantum-dynamical simulation of the protonated water dimer II: Infrared spectrum and vibrational dynamics. *J. Chem. Phys.* **127**, 184303 (2007).
[55] Vendrell, O., Brill, M., Gatti, F., Lauvergnat, D. & Meyer, H.-D. Full dimensional (15D) quantum-dynamical simulation of the protonated water dimer III: mixed Jacobi-valence parametrization and benchmark results for the zero-point energy, vibrationally excited states and infrared spectrum. *J. Chem. Phys.* **130**, 234305 (2009).
[56] Vendrell, O., Gatti, F. & Meyer, H.-D. Full dimensional (15D) quantum-dynamical simulation of the protonated water dimer IV: Isotope effects in the infrared spectra of $D(D_2O)_2^+$, $H(D_2O)_2^+$ and $D(H_2O)_2^+$ isotopologues. *J. Chem. Phys.* **131**, 034308 (2009).
[57] Ndong, M. *et al.* Automatic computer procedure for generating exact and analytical kinetic energy operators based on the polyspherical approach: General formulation and removal of singularities. *J. Chem. Phys.* **139** (2013).
[58] Ndong, M. *et al.* Automatic computer procedure for generating exact and analytical kinetic energy operators based on the polyspherical approach. *J. Chem. Phys.* **136** (2012).
[59] Joubert-Doriol, L. *et al.* Suitable coordinates for quantum dynamics: Applications using the multiconfiguration time-dependent hartree (mctdh) algorithm. *Computational and Theoretical Chemistry* **990**, 75–89 (2012).
[60] Iung, C. & Gatti, F. Polyspherical parametrization of an n-atom system: Principles and applications. *International Journal of Quantum Chemistry* **106**, 130–151 (2005).

[61] Gatti, F. Vector parametrization of the n-atom problem in quantum mechanics. iii. separation into two subsystems: Application to nh3. *J. Chem. Phys.* **111**, 7225–7235 (1999).
[62] Gatti, F., Iung, C., Menou, M. & Chapuisat, X. Vector parametrization of the n-atom problem in quantum mechanics. ii. coupled-angular-momentum spectral representations for four-atom systems. *J. Chem. Phys.* **108**, 8821–8829 (1998).
[63] Gatti, F. *et al.* Vector parametrization of the n-atom problem in quantum mechanics. i. jacobi vectors. *J. Chem. Phys.* **108**, 8804–8820 (1998).
[64] Schran, C., Behler, J. & Marx, D. Automated Fitting of Neural Network Potentials at Coupled Cluster Accuracy: Protonated Water Clusters as Testing Ground. *J. Chem. Theory Comput.* **16**, 88–99 (2020). URL `https://pubs.acs.org/doi/10.1021/acs.jctc.9b00805`.
[65] Schran, C., Brieuc, F. & Marx, D. Transferability of machine learning potentials: Protonated water neural network potential applied to the protonated water hexamer. *J. Chem. Phys.* **154**, 051101 (2021).
[66] Beckmann, R., Brieuc, F., Schran, C. & Marx, D. Infrared spectra at coupled cluster accuracy from neural network representations. *J. Chem. Theory Comput.* **18**, 5492–5501 (2022). URL `https://pubs.acs.org/doi/full/10.1021/acs.jctc.2c00511http://arxiv.org/abs/2202.00303`.
[67] Hutter, J., Iannuzzi, M., Schiffmann, F. & VandeVondele, J. CP2K: atomistic simulations of condensed matter systems. *WIREs Computational Molecular Science* **4**, 15–25 (2014). URL `https://onlinelibrary.wiley.com/doi/abs/10.1002/wcms.1159`.
[68] (2020). CP2K: Open Source Molecular Dynamics; https://www.cp2k.org/.
[69] Brieuc, F., Dammak, H. & Hayoun, M. Quantum Thermal Bath for Path Integral Molecular Dynamics Simulation. *J. Chem. Theory Comput.* **12**, 1351–1359 (2016).
[70] Schran, C., Brieuc, F. & Marx, D. Converged Colored Noise Path Integral Molecular Dynamics Study of the Zundel Cation Down to Ultralow Temperatures at Coupled Cluster Accuracy. *J. Chem. Theory Comput.* **14**, 5068–5078 (2018).
[71] Mendive-Tapia, D., Meyer, H.-D. & Vendrell, O. Optimal mode combinations in the Multiconfiguration Time-Dependent Hartree method through multivariate statistics: Factor analysis and hierarchical clustering. *J. Chem. Theory Comput.* **19**, 1144 (2023).
[72] Székely, G. J., Rizzo, M. L. & Bakirov, N. K. Measuring and testing dependence by correlation of distances. *The Annals of Statistics* **35** (2007).
[73] Schröder, M. Transforming high-dimensional potential energy surfaces into a canonical polyadic decomposition using monte carlo methods. *J. Chem. Phys.* **152**, 024108 (2020).
[74] Schröder, M. Transforming high-dimensional potential energy surfaces into a canonical polyadic decomposition using Monte Carlo methods. *J. Chem. Phys.* **152**, 024108 (2020).
[75] Marx, D., Chandra, A. & Tuckerman, M. E. Aqueous Basic Solutions: Hydroxide Solvation, Structural Diffusion, and Comparison to the Hydrated Proton. *Chem. Rev.* **110**, 2174–2216 (2010).
[76] Marx, D. & Hutter, J. *Ab initio molecular dynamics: Basic theory and advanced methods* (Cambridge University Press, Cambridge, 2009).
[77] Hammer, B., Hansen, L. B. & Nørskov, J. K. Improved adsorption energetics within density-functional theory using revised perdew-burke-ernzerhof functionals. *Phys. Rev. B* **59**, 7413 (1999).
[78] Grimme, S., Antony, J., Ehrlich, S. & Krieg, H. A consistent and accurate ab initio parametrization of density functional dispersion correction (dft-d) for the 94 elements h-pu. *J. Chem. Phys.* **132**, 154104 (2010).
[79] Forster-Tonigold, K. & Groß, A. Dispersion corrected rpbe studies of liquid water. *J. Chem. Phys.* **141**, 064501 (2014).
[80] Goedecker, S., Teter, M. & Hutter, J. Separable dual-space gaussian pseudopotentials. *Phys. Rev. B* **54**, 1703 (1996).
[81] Hartwigsen, C., Gœdecker, S. & Hutter, J. Relativistic separable dual-space gaussian pseudopotentials from h to rn. *Phys. Rev. B* **58**, 3641 (1998).
[82] Krack, M. Pseudopotentials for h to kr optimized for gradient-corrected exchange-correlation functionals. *Theor. Chem. Acc.* **114**, 145–152 (2005).
